# Episodic memory retrieval with increasing task demand: associations with age, *APOE4* genotype and Alzheimer’s disease pathology

**DOI:** 10.1093/braincomms/fcag375

**Published:** 2026-10-07

**Authors:** Finja Askevold, Beate Schumann-Werner, Niklas Behrenbruch, Svenja Schwarck, Eóin N Molloy, Jonathan E Peelle, Ryan M O’Leary, Arthur Wingfield, Gusalija Behnisch, Constanze Seidenbecher, Björn H Schott, Bárbara Morgado, Hermann Esselmann, Jens Wiltfang, Emrah Düzel, Anne Maass, Larissa Fischer

**Affiliations:** German Center for Neurodegenerative Diseases (DZNE), Magdeburg 39120, Germany; Faculty of Natural Sciences, Otto von Guericke University, Magdeburg 39106, Germany; German Center for Neurodegenerative Diseases (DZNE), Magdeburg 39120, Germany; Institute of Cognitive Neurology and Dementia Research, Otto von Guericke University, Magdeburg 39120, Germany; German Center for Neurodegenerative Diseases (DZNE), Magdeburg 39120, Germany; Faculty of Natural Sciences, Otto von Guericke University, Magdeburg 39106, Germany; German Center for Neurodegenerative Diseases (DZNE), Magdeburg 39120, Germany; Institute of Cognitive Neurology and Dementia Research, Otto von Guericke University, Magdeburg 39120, Germany; German Center for Neurodegenerative Diseases (DZNE), Magdeburg 39120, Germany; Division of Nuclear Medicine, Department of Radiology & Nuclear Medicine, Faculty of Medicine, Otto von Guericke University, Magdeburg 39120, Germany; Department of Communication Sciences and Disorders, Northeastern University, Boston 02115, MA, USA; Department of Psychology, Northeastern University, Boston 02115, MA, USA; Department of Communication Sciences and Disorders, Northeastern University, Boston 02115, MA, USA; Department of Psychology, Northeastern University, Boston 02115, MA, USA; Department of Psychology and Volen National Center for Complex Systems, Brandeis University, Waltham 02453, MA, USA; Department of Psychology and Volen National Center for Complex Systems, Brandeis University, Waltham 02453, MA, USA; Leibniz Institute for Neurobiology (LIN), Magdeburg 39118, Germany; Leibniz Institute for Neurobiology (LIN), Magdeburg 39118, Germany; Leibniz Institute for Neurobiology (LIN), Magdeburg 39118, Germany; Department of Psychiatry and Psychotherapy, University Medical Center, Georg-August University, Göttingen 37073, Germany; German Center for Neurodegenerative Diseases (DZNE), Göttingen 37075, Germany; Department of Psychiatry and Psychotherapy, University Medical Center, Georg-August University, Göttingen 37073, Germany; German Center for Neurodegenerative Diseases (DZNE), Göttingen 37075, Germany; Department of Psychiatry and Psychotherapy, University Medical Center, Georg-August University, Göttingen 37073, Germany; Department of Psychiatry and Psychotherapy, University Medical Center, Georg-August University, Göttingen 37073, Germany; German Center for Neurodegenerative Diseases (DZNE), Göttingen 37075, Germany; German Center for Neurodegenerative Diseases (DZNE), Magdeburg 39120, Germany; Institute of Cognitive Neurology and Dementia Research, Otto von Guericke University, Magdeburg 39120, Germany; German Center for Neurodegenerative Diseases (DZNE), Magdeburg 39120, Germany; Faculty of Natural Sciences, Otto von Guericke University, Magdeburg 39106, Germany; German Center for Neurodegenerative Diseases (DZNE), Magdeburg 39120, Germany; Faculty of Natural Sciences, Otto von Guericke University, Magdeburg 39106, Germany

**Keywords:** aging, *APOE4*, episodic memory, retrieval difficulty, task demand

## Abstract

Episodic memory declines early with ageing, reflecting reduced neural resources and diminished memory specificity. However, few studies have created a cognitive challenge with multiple levels of task demand to investigate this early, subtle decline. Furthermore, it is unclear whether the genetic Alzheimer’s disease risk factor *APOE4* and early Alzheimer’s pathology constrain the neural resources required to cope with increasing task demands.

In this preregistered behavioural study named EMCOMP (Episodic Memory & COMPensation), we conducted a sentence-based episodic memory retrieval task where we manipulated retrieval difficulty by varying the required degree of memory specificity, thereby increasing task demands on episodic retrieval with five demand levels at recognition (novel foils, ‘old’ target sentences and three lure levels) and two demand levels at free and cued recall (gist and details). We collected data from 100 cognitively unimpaired adults [37 young (mean age 24 years), 63 older (mean age 72 years)] with additional neuropsychological testing and blood-based measures available for the older group. First, we investigated differences in EMCOMP episodic memory retrieval accuracy and confidence between young and older adults in linear mixed-effects models. Second, we investigated differences associated with the *APOE4* genotype and plasma-derived Alzheimer’s pathology. Third, we assessed correlations between EMCOMP recognition and recall and established cognitive tests. Young adults showed higher recognition accuracy and confidence as well as a higher recall score and a lower recall error rate compared to older adults. As target-lure similarity increased, there was a steeper decline in recognition accuracy and a steeper increase in high-confidence errors in older compared to younger adults and in older *APOE4* carriers compared to older non-carriers. However, we found no associations with Alzheimer’s pathology. EMCOMP performance was positively correlated with established cognitive tests.

Our study demonstrates age- and *APOE4*-related differences in episodic memory retrieval under increasing retrieval difficulty—differences that may not be detectable in paradigms with only one or two levels of variation. By manipulating sentence-based retrieval specificity, we provide a novel approach to detect subtle cognitive deficits and potential functional compensation in cognitively unimpaired older adults with risk factors or early Alzheimer’s pathology. Future research should extend this work to more diverse samples and combine behavioural assessment with functional magnetic resonance imaging (MRI) to examine underlying brain activation patterns and functional connectivity.

## Teaser

Askevold et al. introduce a sentence-based episodic-memory task. They report reduced accuracy and more high-confidence errors with increasing retrieval difficulty in older compared to young adults and older *APOE4* carriers compared to non-carriers. By manipulating the required memory specificity, the approach enables detection of subtle cognitive deficits and potential compensation.

## Graphical Abstract

**Graphical Abstract:**
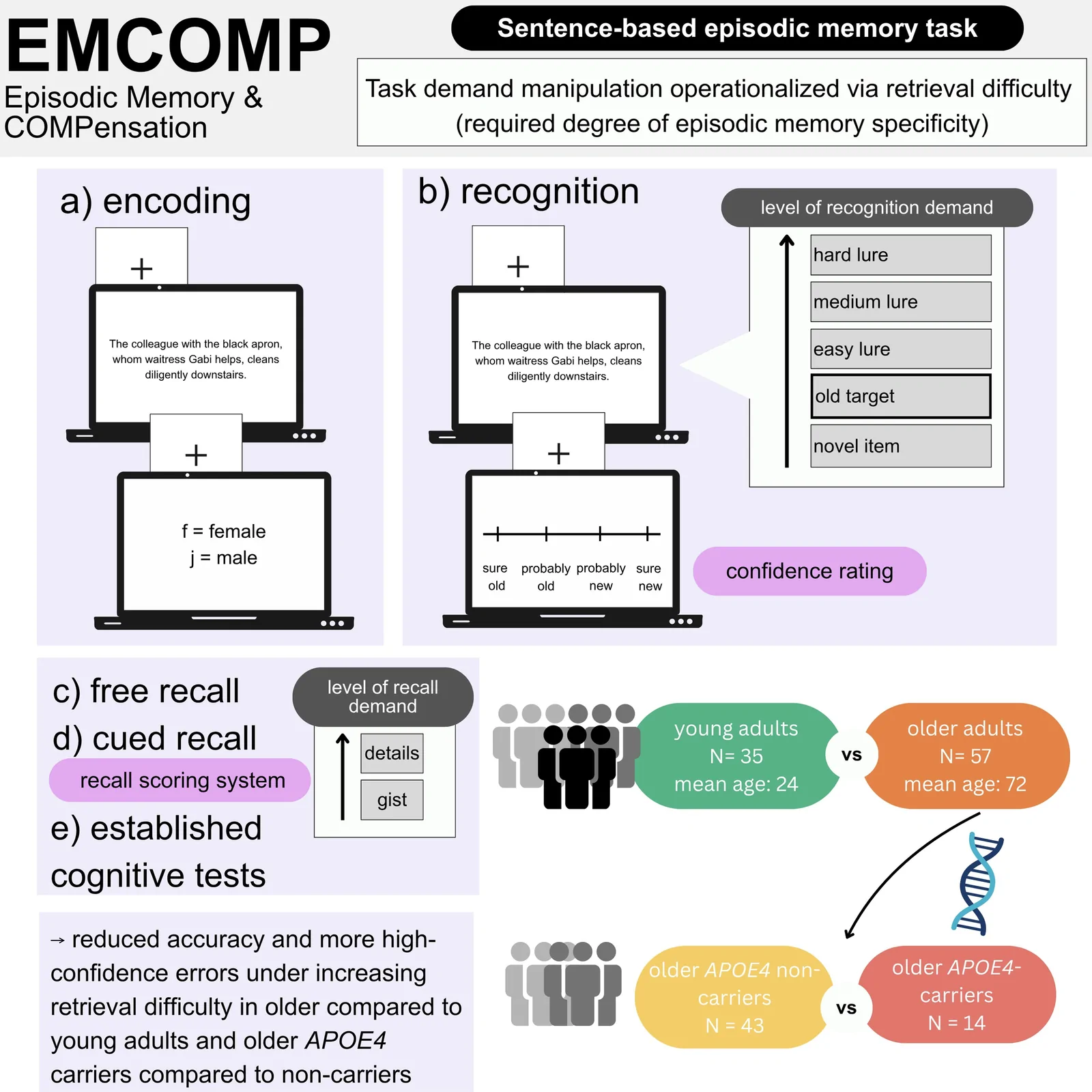

## Introduction

Cognitive decline occurs with ageing, especially when Alzheimer’s disease risk factors or early Alzheimer’s pathology are present.^1,2^ Episodic memory is among the cognitive functions that show reliable age-related decline,^3,4^ accompanied by a decline in working memory capacity and processing speed,^5,6^ whereas language processing^7^ and vocabulary^2^ tend to be preserved. Importantly, cognitive functions rarely operate in isolation; rather, they interact dynamically. For instance, higher working memory capacity and vocabulary can support episodic memory retrieval in older adults.^8-10^ Beyond age-related changes such as synaptic alterations,^11,12^ most cognitively unimpaired older adults harbour^13,14^ tau tangles within the medial temporal lobe,^15,16^ one hallmark of Alzheimer’s disease. Some cognitively normal older adults also harbour neocortical amyloid-beta (Aβ) plaques,^17,18^ which is often associated with unfavourable cognitive trajectories,^19^ rendering the detection of early subtle cognitive decline an important aim.

One of the major risk factors for sporadic Alzheimer’s disease is the Apolipoprotein-E4 (*APOE4*) genotype,^20,21^ which is strongly correlated with Aβ accumulation.^22,23^ While some studies report subtle early cognitive deficits in *APOE4* carriers already in midlife,^24,25^ most studies did not find *APOE*-related cognitive or neural differences in middle-aged adults (for reviews, see^26,27^). In cognitively unimpaired older *APOE4* carriers, deficits in visual mnemonic discrimination^28^ and episodic memory retrieval of words^29^ have been reported, whereas another study reported no effects of *APOE4* on object-location binding.^30^ In older adults with subjective cognitive decline (SCD), older *APOE4* carriers showed a steeper decline in general cognition compared to cognitively unimpaired *APOE4* carriers.^31^ These mixed results underscore the importance of identifying subtle early cognitive decline in vulnerable individuals before a decline in general cognition becomes apparent and further accelerates. Furthermore, in the era of Alzheimer’s disease treatment,^32-34^ early detection of individuals in need of monitoring and medical intervention is key. An important question to answer is therefore how to detect early subtle cognitive deficits to identify these individuals who are still cognitively unimpaired in standard neuropsychological testing.

Theories regarding intact cognition and brain function despite the loss of neural resources, e.g. due to Alzheimer’s pathology, are gathered under the umbrella term of resilience.^35,36^ One mechanism of resilience is functional compensation: the short-term recruitment of additional neural resources in response to higher task demands. Task demand influences cognitive performance, with more demanding task conditions creating a greater cognitive challenge that needs to be met with the available neural resources.^37-40^ During a cognitive task, short-term changes in brain network activity, e.g. more recruitment of domain-general cognitive control brain networks,^41,42^ may be required to meet the current task demands. When fewer neural resources are available due to age- or Alzheimer’s disease-related processes, more effort is needed to meet task demands at a lower demand level,^37,43^ and a failure of compensatory mechanisms could occur earlier.^44^ For an overview of theories and a discussion of compensatory versus detrimental effects, see.^45^ A resulting question is how to empirically investigate these theoretically proposed mechanisms of compensation related to episodic memory retrieval task demand.

One experimental approach is to gradually increase task demands across multiple levels to create a stepwise increase in cognitive challenge.^46-49^ Few studies to date have, however, used a manipulation of retrieval-task demand to investigate episodic memory.^50^ Furthermore, ageing studies often do not consider Alzheimer’s disease risk factors and pathology in cognitively unimpaired older adults, which is crucial for understanding processes related to ageing.^51-53^ To address this research gap, we adapted a behavioural task previously used for immediate retrieval^48,54,55^ to manipulate retrieval difficulty by varying the required degree of memory specificity, which we expected to increase demands on episodic retrieval. Within the EMCOMP (Episodic Memory & COMPensation) study, we first assessed retrieval performance across five recognition and two recall demand levels in young adults and cognitively unimpaired older adults. Second, we assessed the influence of the *APOE4* genotype and plasma-based Alzheimer’s pathology burden on retrieval performance in older adults. Third, we assessed the relation to performance on established cognitive tests in older adults.

We hypothesized that (i) retrieval accuracy declines with increasing task demand, (ii) older compared to younger adults, *APOE4* carriers compared to non-carriers and individuals with more Alzheimer’s pathology show lower recognition accuracy, recall less correct information and show a steeper decline in performance with higher task demand and (iii) older adults’ confidence in their response follows a U-shape, i.e. an initial decrease in confidence (in the correct response) as demand increases, followed by an increase in confidence (in the incorrect response) as demand increases further. In individuals with the *APOE4* genotype and higher Alzheimer’s pathology burden, these effects become apparent even at lower demand levels.

## Materials and methods

### Sample

We collected data from 37 young and 63 older adults. All participants were native German speakers, self-reported as White and of European ancestry, had normal or corrected to normal vision and reported no history of neurological or mental disorders including dyslexia. All participants were required to reach at least 80% accuracy in the encoding cover task (see section ‘Encoding session’) to ensure attention on the task.

Young adults were primarily recruited via online advertisement and were predominantly students at the Otto-von-Guericke University Magdeburg and the University of Applied Sciences Magdeburg-Stendal. Older adults were recruited from the participant pool of the Z03 project, which is part of the Collaborative Research Centre (CRC) 1436 ‘Neural Resources of Cognition’ (see^56^ for details).

In short, older participants were over the age of 60 and cognitively unimpaired with scores within their age range of the CERAD-Plus (Consortium to Establish a Registry for Alzheimer’s Disease) Neuropsychological Assessment Battery. Additionally, they had no major age-related illnesses. After excluding eight participants (two young adults did not meet the inclusion criteria, four older adults misunderstood the task, two older adults did not reach the threshold for the encoding cover task), the final sample consisted of 35 young (24 ± 3 years, 17 female) and 57 older adults (72 ± 0 years, 31 female, 14 *APOE4* carriers). *APOE4* carriers and non-carriers did not differ significantly in demographic variables (see Supplementary Methods 1 for more information regarding the *APOE* genotype). Demographic details and additional information can be found in Table 1.

**Table 1:** Description of the sample

Age group	*N*	Age in years	Female sex	Education in years	Vocabulary score	Working memory capacity	*APOE4* carrier	Alzheimer’s disease pathology (AT-Term)
Young adults	35	23.8 (2.9) 18–30	17 (49%)	16.5 (2.2)	99.0 (4.7)	0.9 (0.5)	—	—
Older adults	57	72.0 (7.2) 62–89	31 (54%)	15.6 (2.4)	112.0 (9.3)	0.6 (0.8)	14 (25%)	1.45 (1.01)

Mean (standard deviation) or number (percentage) of participants. Age denotes age at baseline; age range is additionally provided. All participants identified as either female or male. *APOE* genotype: ε3/ε3 = 0.68 (*n* = 39), ε3/ε4 = 0.16 (*n* = 9), ε3/ε2 = 0.05 (*n* = 3), ε4/ε4 = 0.05 (*n* = 3), ε4/ε2 = 0.04 (*n* = 2), ε2/ε2 = 0.02 (*n* = 1). For the vocabulary score, Mehrfachwahl-Wortschatz-Intelligenztest (MWT-B) normative values were used. Working memory capacity was assessed using a change detection task. For Alzheimer’s disease pathology, the AT-Term 1/(Aβ_1–42_/Aβ_1–40_) *P-tau_217_ was used.

In our pre-registration,^57^ we planned to recruit 35 young and 35 older adults for age-group comparisons for a Bachelor’s Thesis, resulting in a total of 70 participants. We estimated this required sample size using G*Power for a multiple linear regression with recall performance as the dependent variable and age group, recall demand, sex, vocabulary score and working memory score as predictors. Assuming a medium-to-large effect size (*f*^2^ = 0.20) for an age group effect, an α-level of 0.05 and a desired power of 0.80, the analysis yielded a required sample size of 70 participants. In addition, we predetermined to continue collecting and processing data from older adults for the analysis of *APOE4* group and Alzheimer’s pathology until October 2025. This pre-specified endpoint was based on available personnel resources, and no formal power calculation was performed for the following analyses involving *APOE4* genotype and Alzheimer's pathology.

All study procedures and experimental protocols were approved by the Ethics Committee of the Medical Faculty, Otto-von-Guericke University Magdeburg (200/19) and were carried out in accordance with the Declaration of Helsinki. All participants provided written informed consent.

### Episodic memory retrieval task-demand manipulation

#### Stimuli

The experiment included three main sessions: encoding, recognition and recall. For each participant, an individual encoding item (i.e. sentence) list was created with the same 75 sentences in a randomized order. Furthermore, one encoding-practice list for all participants was created using five sentences. For the recognition task, a list of 93 sentences was constructed including sentences of all five recognition-demand levels from easy to hard: 18 completely novel foils, 21 ‘old’ target sentences (were presented during encoding), 18 easy lures, 18 medium lures and 18 hard lures. The allocation of original sentences to recognition demand level was semi-randomized over all three sentence types. Recognition demand levels were based on previous work.^58,59^ Furthermore, a recognition-practice list was created with retrieval-demand manipulation of the encoding practice sentences. Stimuli are openly available on the OSF (https://doi.org/10.17605/OSF.IO/E4MC9). In the following, the construction of base and lure items is described.

Of all 93 written German sentences, 75 sentences were first seen at encoding, and the remaining 18 were first seen at recognition as novel foils. All sentences were similar in length (approximately 13 words) and included unique content and words. The construction of the stimuli was based on previous work, where meaningful 10-word sentences were used to investigate syntactic working memory load and immediate recall.^46,48^ In this study, the sentences from DeCaro and colleagues^48^ were translated from English into German. Then, the sentences were extended with additional information, including social and spatial relations, to provide both gist-level and detailed content for later retrieval. All sentences underwent an unpublished stimuli rating in which eight young and six older adults (*N* = 14) read the sentences without time restrictions and assessed them on a nine-point scale (from −4 to 4) in the following categories: emotion, schema congruency, comprehensibility, word frequency and memorability. This rating was conducted to ensure that the sentences were approximately similar within their respective categories when no challenge due to a time restriction was present. The rating resulted in five sentences being conspicuous in terms of schema congruency, so they were altered to be more congruent.

Three different sentence types with a total of 31 subject-relative sentences with a long agent-action gap (subject-long), 31 object-relative sentences with a short gap (object-short) and 31 object-relative sentences with a long gap (object-long) were used (for details, see^48^; e.g. see Table 2). Object-relative syntactic structures are cognitively more challenging compared to syntactically simpler subject-relative structures.^48^ This design was implemented for future use of the task in the MRI scanner, where different levels of encoding-demand will be investigated in relation to brain activation during encoding. In this behavioural pre-study, the encoding session encompassed a cover task (see section ‘Encoding session’). Use of names, sex and plural form was balanced across the three sentence types. Sentence types were balanced across encoding, ‘old’ target and novel items.

**Table 2:** Examples of base items

Sentence type	Example
Subject-long	Die Mütter aus dem dritten Stock, die Kai zum Fußballspiel einladen, sind sympathisch. The moms from the third floor who invite Kai to the football match are likeable.
Object-short	Der junge Elektriker aus der Nachbargemeinde, dem Olivia hilft, ist endlich selbständig. The young electrician from the neighboring community, whom Olivia helps, is finally self-employed.
Object-long	Die Enkeltochter, der Opa Herbert mit Schmerzen im unteren Rücken vorliest, ist schläfrig. The granddaughter, whom Grandpa Herbert with pain in the lower back reads to, is sleepy.

One example for each of the three sentence types of base stimuli in German, with a literal translation in English below. ‘Object-long’ means an object-relative sentence with a long agent-action gap. For agent-action gap visualization, the agent and the action of the sentence are underlined. Cognitive challenge is increasing from subject-long to object-short to object-long syntaxes.^48^.

For the recognition task, 54 lures (three recognition-demand levels; 18 stimuli per level) were created. As lure items, encoded stimuli were altered, resulting in novel items with varying levels of similarity to the originally encoded items. For the easy lure level, two words were replaced by non-synonymous words (e.g. ‘apron’ replaced by ‘wig’ and ‘diligently’ replaced by ‘lazily’; see Table 3), which changed the meaning of the original sentence. For medium lure level, only one word was replaced by a non-synonymous word (e.g. ‘colleague’ replaced by ‘trainee’), which is, however, part of the same word category so that the content only changed slightly. For the hard lure level, one word was exchanged with a synonym, so the meaning of the sentence remained the same (e.g. ‘black’ replaced by ‘dark’). The full example sentences are provided in Table 3. To check synonyms, the online Oxford Languages English Dictionary^60^ was used. To keep the word frequency of the exchanged words consistent, the online German Dictionary ‘DWDS—Digitales Wörterbuch der Deutschen Sprache’^61^ was used.

**Table 3:** Examples of lure items

Sentence type	Example
Base item	Der Kollege mit schwarzer Schürze, dem Kellnerin Gabi hilft, ist unten beim Putzen fleißig. The colleague with the black apron, whom waitress Gabi helps, cleans diligently downstairs.
Easy lure	Der Kollege mit schwarzer **Perücke**, dem Kellnerin Gabi hilft, ist unten beim Putzen **faul**. The colleague with the black **wig**, whom waitress Gabi helps, cleans **lazily** downstairs.
Medium lure	Der **Praktikant** mit schwarzer Schürze, dem Kellnerin Gabi hilft, ist unten beim Putzen fleißig. The **trainee** with the black apron, whom waitress Gabi helps, cleans diligently downstairs.
Hard lure	Der Kollege mit **dunkler** Schürze, dem Kellnerin Gabi hilft, ist unten beim Putzen fleißig. The colleague with the **dark** apron, whom waitress Gabi helps, cleans diligently downstairs.

Demonstration of the recognition demand manipulation using one base sentence as an example for the creation of all three lure levels in German with a literal translation in English below. Easy lures were created by replacing two words with non-synonym words changing the content of the sentence drastically. For medium lures, one word was replaced to change the content slightly. For hard lures, one word was replaced with a synonym. The replaced words are marked in bold.

#### Encoding session

The encoding session was conducted using PsychoPy version 2023.2.3.^62^ The instruction, the items and the fixation cross were presented in white (RGB: 255,255,255) on a grey background (RGB: 128,128,128). In the intentional encoding session, each participant viewed all 75 target items. Participants were instructed to read each sentence, try to memorize it and indicate via keypress whether the first person mentioned was female or male. The female/male decision task was included as a cover task to ensure that the respective sentence was processed by the participant. However, it was emphasized in the instructions that the focus of the encoding task was to memorize the sentences. Each trial of the encoding task (see Fig. 1) started with a fixation cross that was randomly displayed for 2–6 s. The randomized presentation time of the fixation cross was set to minimize habituation effects and used in light of the planned implementations of the task in the MRI scanner. After the fixation cross, a sentence was presented for seven seconds in which the participants had time to read the sentence thoroughly and remember as much as possible. Seven seconds for encoding were determined in our preregistration based on the self-paced reading speed of young adults (400 ms/word) and older adults (700 ms/word).^63^ For a 13-word sentence, this corresponds to approximately five and nine seconds, respectively. As the aim of the study was to create a cognitive challenge, especially in older adults, the mean combined self-paced reading speed of both age groups was used, which resulted in 7 s. The item presentation was followed by another randomized presentation of the fixation cross, for 2–6 s, followed by the decision task to indicate the gender of the first person mentioned. Participants responded via keypress (F = female, J = male), within a response window of two seconds. One trial took about 15 s. The encoding task, including the practice part of five trials, took about 25 min.

**Figure 1:**
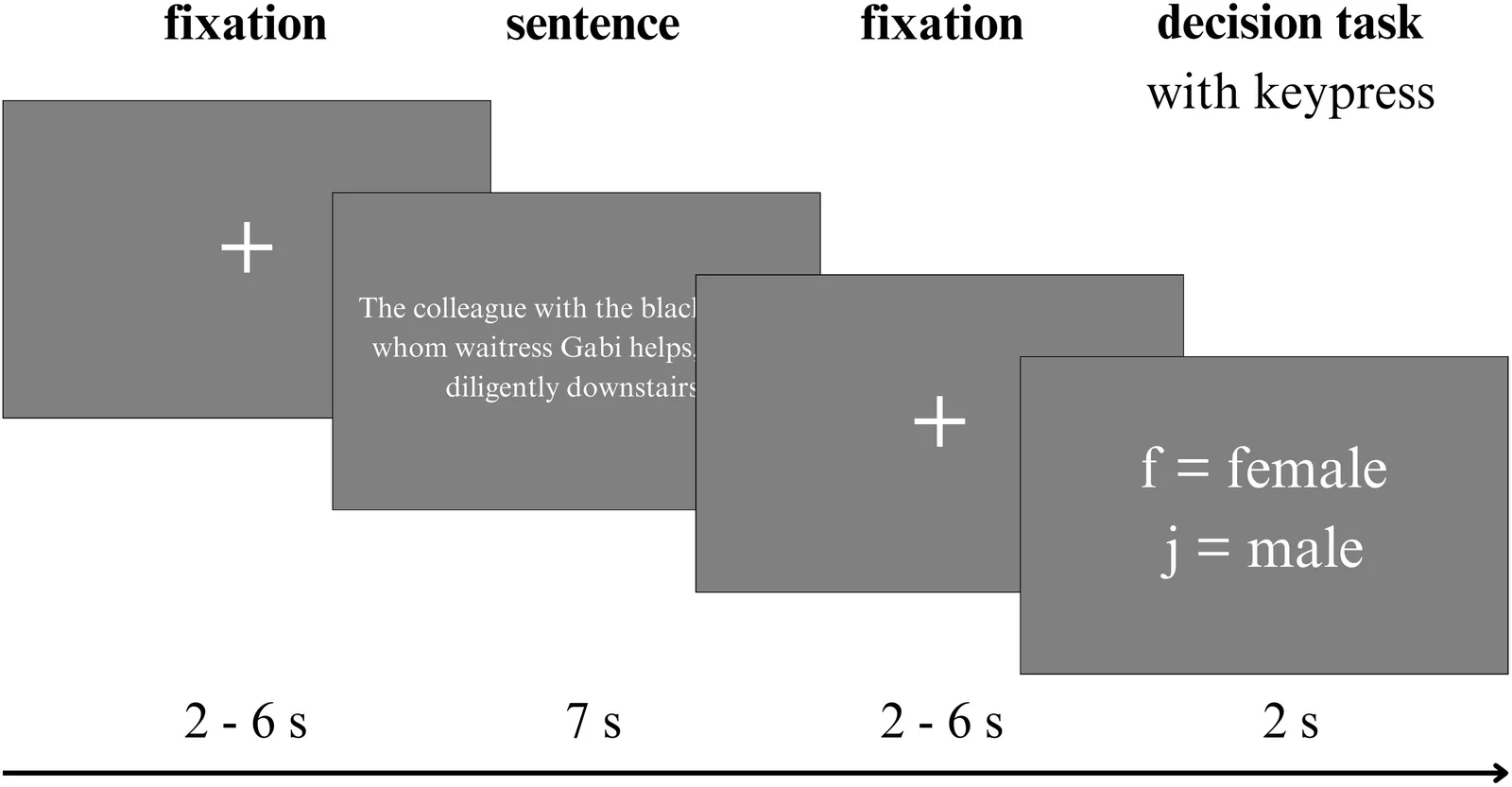
**Procedure of one trial in the encoding task.** This figure shows the procedure of one trial in the encoding task. First, a fixation cross was presented, then the target item (sentence) was presented, followed again by a fixation cross of variable duration. Then the decision task followed to indicate the gender of the first person mentioned. Answers were given via key press.

#### Recognition session

The recognition session started 15 min after the encoding session ended. Within these 15 min, participants watched a silent nature documentary. The same PsychoPy setup was used as for the encoding session. The main task was the identification of ‘old’ target sentences that participants had encoded before, in contrast to lures and novel foils. Participants viewed 93 stimuli, including 21 ‘old’ target sentences, 18 novel foils and 54 lure sentences (18 in each lure level). Participants were instructed to read the displayed sentence and decide whether it was ‘old’ (already seen during encoding) or ‘new’ (lure or novel foil). Additionally, they should indicate their confidence in their categorization of the sentence. Both categorization and confidence were reported in a confidence rating via keypress. The structure of the trials in the recognition task (see Fig. 2) was similar to that of the encoding task. Each trial began with the presentation of the fixation cross for a randomized period of 2–6 s. Then, a sentence was displayed for 7 s. After another presentation of the fixation cross, the confidence rating followed. Participants again had a 2-s time window to give their answer via keypress. They were instructed to press 1 if they were sure they had read the sentence in the encoding task before (= sure old), press 2 if they were unsure but thought the sentence was probably old (= probably old). The same principle applied if they thought the sentence was new: 8 = probably new; 9 = sure new. One trial lasted for about 15 s, followed by the next trial. Similar to the encoding task, there was a practice part before the main recognition task started. The recognition task took about 25 min.

**Figure 2:**
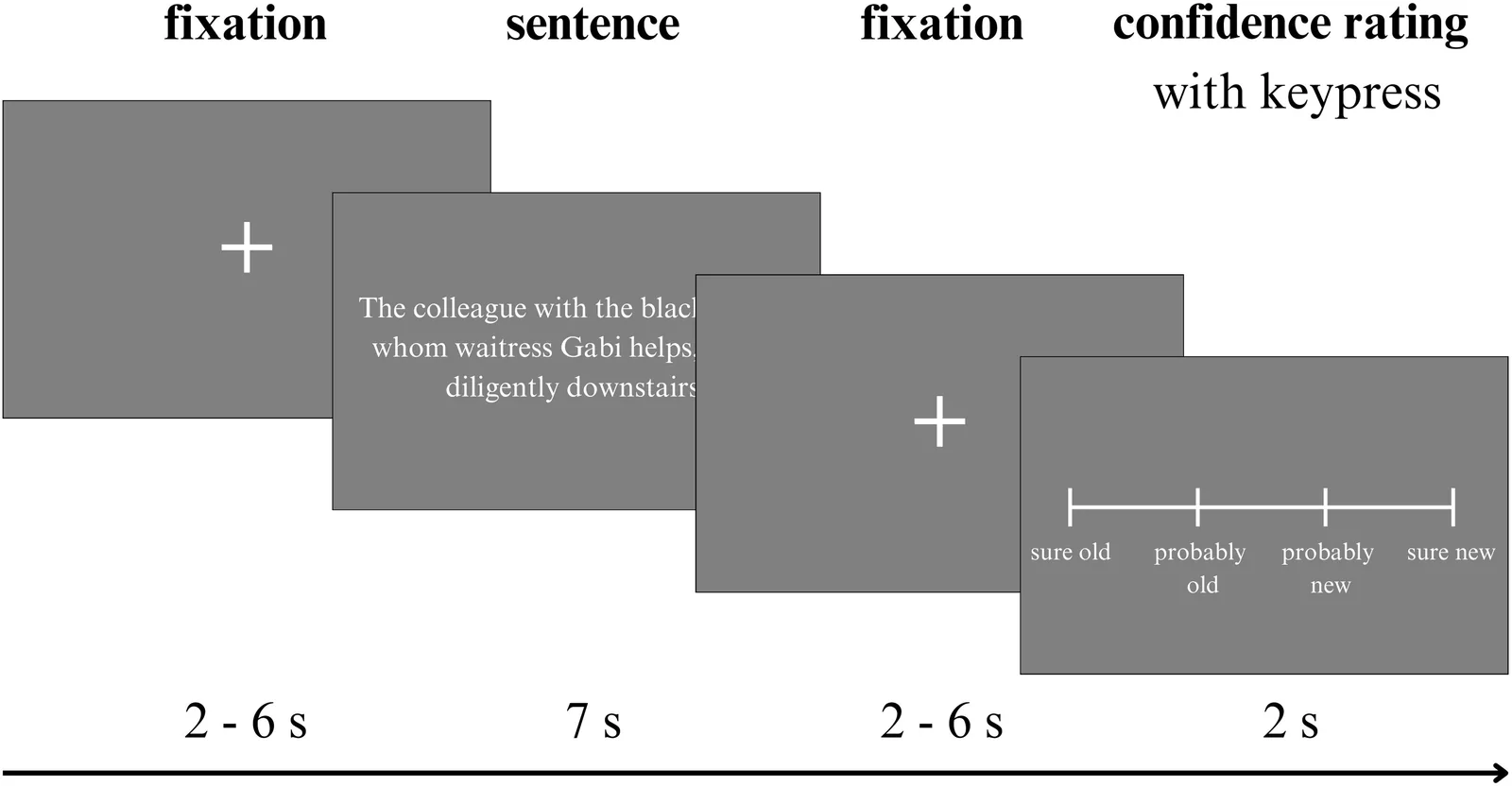
**Procedure of one trial in the recognition task.** This figure shows the procedure of one trial in the recognition task. First, a fixation cross was presented, then the demand manipulated target item was presented (new or old or lure level), before the confidence rating followed, after another fixation cross. Answers were given via key press.

#### Recall session

The recall session was divided into two parts: free recall (approximately 90 min after the start of encoding) and cued recall (approximately 100 min after the start of encoding). In the free recall task, participants were prompted with the question, ‘Which sentences do you remember from the first (encoding) experiment?’. They were encouraged to reproduce everything they remembered, full sentences and phrases or fragments of the sentences were possible. When they had recalled everything they knew, the cued recall followed. The instructor read the first half of every sentence (6 words) out loud as a cue. If participants remembered anything about the second half, they were supposed to complete the sentence. Both recall types were audio-recorded to simplify the evaluation and to ensure that no information was lost. Afterwards, the audio was transferred into text and scored with a recall scoring system (see Supplementary Methods 2 and Supplementary Table 1). Then, a relative recall score was computed by dividing each participant’s obtained recall score by the maximum possible score, assuming complete recall.

#### Cognitive testing

For measuring the participants’ vocabulary, the B Version of the German Mehrfachwahl-Wortschatz-Intelligenztest (MWT-B)^64^ (translated: ‘multiple choice vocabulary intelligence test’) was used. The MWT-B is used to measure the level of crystalline intelligence. Participants are instructed to find the existing German word among four other invented non-existing similar words. The words in the German language originate from colloquial, educational or scientific contexts and become more specific and rare with each row. In total, there are 37 rows with five words each. Z-values were derived using the test manual.

A computer-based change detection task was carried out to measure visual working memory capacity. In this experiment, an open-source version^65^ was used. In the change detection paradigm, participants first see a fixation cross for 1 s, an array of six visual items (squares) of different colours for 0.5 s, another fixation cross as a delay phase for 1 s, and then a single test item (colored square) as probe until keypress. Participants had to decide via keypress whether the position and colour of the probe is similar to the position and colour of one of the presented items.^66^ In this study, the visual working memory capacity *K* was calculated using a standard formula^67,68^: *K* = *N* × (*H*−FA), where *K* is the capacity estimate, *N* is the set-size, *H* is the hit rate (proportion of correct responses in change trials) and FA is the false alarm rate (proportion of incorrect responses in no-change trials).

Scores from neuropsychological assessment of older adults included tests focusing on episodic memory and other cognitive functions. For details, see.^56^ Regarding episodic memory, the Verbal Learning and Memory Test (VLMT)^69^ with 15 words, and the Rey Complex Figure Test and Recognition Trial (RCFT)^70^ with one complex figure were used for delayed free recall and recognition. Furthermore, Logical Memory (LM) II of the Wechsler Memory Scale (WMS)^71^ was used for delayed free recall of stories; a German picture version of the Free and Cued Selective Reminding Test (FCSRT)^72^ provided a sum score of free and cued immediate recall.

Regarding further cognitive functions, the Symbol Digit Modalities Test (SDMT)^73^ was used to assess processing speed. The German Regensburger Wortflüssigkeitstest (RWT),^74^ which includes semantic and phonemic verbal fluency tasks, was used to assess executive functioning. The CERAD-Plus Neuropsychological Assessment Battery^75^ was used to assess general cognition with a composite score including: Mini-Mental State Examination (MMSE), verbal and phonemic fluency, a short form of the Boston Naming Test, 10-word list learning and delayed recall and recognition, constructional praxis of figures and delayed recall and the Trail Making Test (TMT) version A and B.

#### Assessment of Alzheimer’s disease plasma biomarkers

Plasma Aβ_1–40_, Aβ_1–42_ and p-tau_217_ were determined using Lumipulse assays in older adults. For details, see.^56^ To investigate a composite measure of Alzheimer’s disease pathology, the AT-Term = 1/(Aβ_1–42_/Aβ_1–40_) * p-tau_217_ was used.

#### *APOE* genotyping

All included older participants were genotyped for *APOE* via a polymerase chain reaction-based approach followed by restriction fragment length polymorphism analysis. For details, see.^56^  *APOE4* carriers were defined as individuals with one or two *APOE4* alleles. *APOE4* non-carriers were defined as individuals without an *APOE4* allele.

### Study design

A preregistration of *a priori* hypotheses and methods was uploaded on the Open Science Framework (OSF)^57^ before data collection was completed and before data were compiled and accessed. For investigating how recognition demand (five levels: novel items, ‘old’ target items, easy lure, medium lure and hard lure) and age group (two levels: young adults and older adults), or *APOE4* group (two levels: carrier and non-carrier) and the AT-Term (continuous measure) in older adults only, influence recognition performance, the dependent variables were accuracy and confidence. In the analysis including only older adults, age was treated as a continuous predictor. Recognition accuracy refers to the number of correct trials relative to all trials per demand level, and confidence refers to the subjective assessment of one’s own success in categorizing a sentence correctly. For investigating effects on recall performance, recall demand (two levels: gist, detail) was examined in relation to age group or *APOE4* group and Alzheimer’s pathology. The dependent variables were recall score and the recall error rate (swaps and add-ons). They all resulted from the recall scoring system (see Supplementary Methods 2 and Supplementary Table 1).

### Statistical analysis

Statistical analysis was conducted using R Version 4.3.1 (RStudio Team, Boston, MA, USA) implemented in RStudio (Version 2024.12.1). The R code used for analyses is publicly available (https://github.com/fislarissa/EMCOMP_demand). Multiple linear regression and linear mixed-effects models (LMMs) with a random intercept for participant were used. In all models, sex, vocabulary score and working memory capacity were included as covariates as predefined in the preregistration based on a previous study.^10^ Education was not used as a covariate, as a further planned analysis aims at investigating education as a proxy for cognitive reserve. The Z03 measurements in older adults were obtained before the EMCOMP measurements (median 15 months, range 3 to 38 months). Including the individual time difference in the models did not change the results. The statistical level of significance was set at *P* < 0.05 for all analyses. Regarding model requirements, it was ensured that the assumption of homoscedasticity was met using the Breusch-Pagan test and that no multicollinearity was present with a variance inflation factor (VIF) below 5. Furthermore, the assumption of a normal distribution of residuals was assessed using quantile-quantile (QQ) plots. Multiple comparisons were controlled using False-discovery-rate (FDR) correction according to the Benjamini-Hochberg procedure.

First, we investigated the influence of recognition-demand level and the interaction of recognition-demand level by age group on recognition accuracy in an LMM (*Accuracy* ∼ *Recognition-demand level (linear) × Age group*  *+*  *Sex*  *+*  *Vocabulary*  *+*  *Working memory capacity)*. We then investigated the influence thereof on recognition confidence, including a U-shape association of confidence across demand levels (*Confidence* ∼ *Recognition-demand level (linear) × Age group*  *+*  *Recognition-demand level (U-shape) × Age group*  *+*  *Sex*  *+*  *Vocabulary*  *+*  *Working memory capacity)*, and further investigated the influence of accuracy on recognition confidence (*Confidence* ∼ *Recognition-demand level (linear) × Age group × Accuracy*  *+*  *Sex*  *+*  *Vocabulary*  *+*  *Working memory capacity).*

Then we investigated the influence of recall-demand level and the interaction of recall-demand level by age group on recall score (*Recall score* ∼ *Recall-demand level (linear) × Age group*  *+*  *Sex*  *+*  *Vocabulary*  *+*  *Working memory capacity)* and investigated recall error rate (*Recall error rate* ∼ *Age group*  *+*  *Sex*  *+*  *Vocabulary*  *+*  *Working memory capacity).* As the residuals were not normally distributed, a log-transformation was applied to the error rates. For interpretation of the results on the original scale, the regression coefficients (β) were additionally exponentiated [exp(β)].

Second, only in older adults, we repeated the described models with the *APOE4* group instead of the age group. Additional predictors were age at baseline and the AT-Term. Exploratively, we repeated these models to investigate VLMT and RCFT recognition performance instead of EMCOMP performance.

Third, only in older adults, we conducted Pearson correlation analysis for (i) recognition accuracy, using the EMCOMP corrected hit rate [hits (categorizing ‘old’ target items correctly as old)—false alarms (categorizing novel items incorrectly as old)] and corrected hit rate for the episodic memory tests VLMT and RCFT; (ii) recall, using the EMCOMP recall score across both demand levels and the delayed recall scores of the episodic memory tests VLMT, RCFT, WMS and immediate recall score of the FCSRT; (iii) established cognitive tests, using the EMCOMP corrected hit rate and EMCOMP recall score across both demand levels and test scores for processing speed (SDMT), executive functioning (RWT) and general cognition (CERAD).

## Results

### Retrieval performance in young and older adults

First, we investigated the effect of recognition-demand level and the interaction of recognition-demand level by age group on recognition accuracy (see Table 4 for descriptive values, Fig. 3A and Supplementary Table 2). We included sex, vocabulary and working memory capacity as covariates, as in all following LMMs. The model revealed that in both age groups, accuracy decreased with increasing recognition-task demand [β = −0.63 (95% CI −0.73, −0.53), *t*(360) = −12.71, *P* < 0.001] and accuracy was highest for identifying novel foils and lowest for discriminating hard lures. Older adults performed worse than young adults across all recognition-demand levels [β = −0.35 (95% CI −0.52, −0.28), *t*(360) = −3.99, *P* < 0.001]. In addition, there was an interaction effect of recognition demand by age group [β = −0.13 (95% CI −0.25, −0.00), *t*(360) = −2.01, *P* = 0.045, Cohen’s *f*^2^ = 0.01], with older adults’ accuracy showing a steeper decline with increasing recognition-task demand. Exploratory post-hoc analysis of the interaction effect with recognition-demand level as a factor revealed a significant difference between age groups for the third recognition-demand level (easy lures) [*t*(434) = 5.179, *P* < 0.001, SE = 0.035] and the fifth recognition-demand level (hard lures) [*t*(224) = 3.242, *P* = 0.001, SE = 0.035] using emmeans.

**Figure 3:**
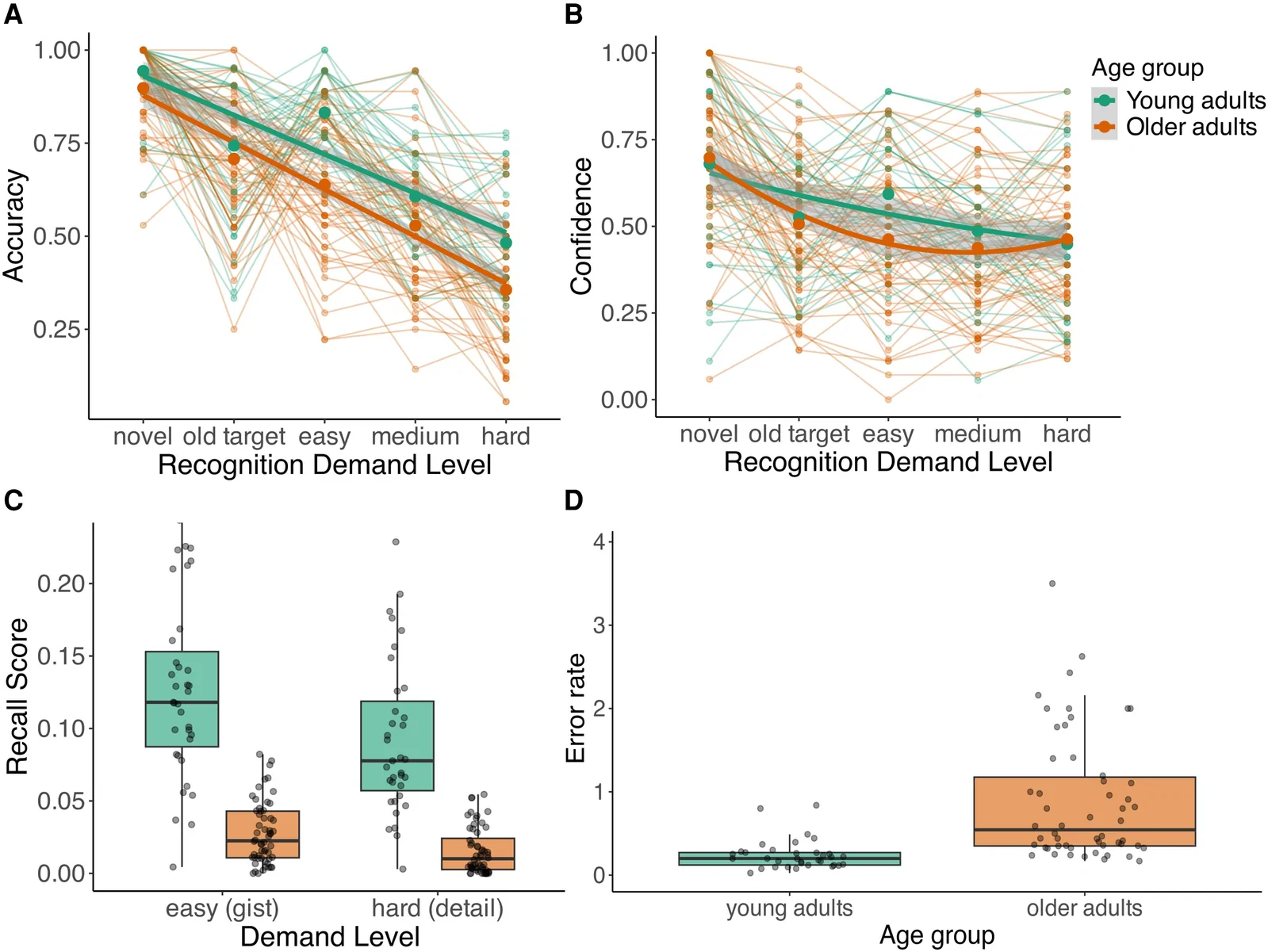
**Recognition-task performance for young and older adults.** (**A**) Accuracy across recognition-task demand levels for young (green lines; *N* = 35) and older adults (orange lines; *N* = 57) was assessed via a linear mixed model. There was an interaction effect of recognition demand by age group [β = −0.13, *t*(360) = −2.01, *P* = 0.045]. Presented are group means (bold dots), smoothed group mean trajectories (bold lines) and individual trajectories (fine lines). (**B**) Confidence across recognition-task demand levels for young (green lines; *N* = 35) and older adults (orange lines; *N* = 57) was assessed via a linear mixed model. There was an interaction effect of recognition-task demand (U-shape) by age group [β = 0.24, *t*(368) = 3.11, *P* = 0.002]. Presented are group means (bold dots), smoothed group mean trajectories (bold lines) and individual trajectories (fine lines). (**C**) Recall score for both recall-demand level (gist and detail) ranging from 0 to 1 for young (green boxes; *N* = 35) and older adults (orange boxes; *N* = 56) was assessed via a linear model. Performance decreased with increasing recall-task demand [β = −0.29, *t*(91) = −13.00, *P* < 0.001]. A score of 0.10 means that 10% of the information the participant was exposed to was recalled. The median is shown as a bold black horizontal line and the 25th and 75th percentile as black horizontal lines below and above, respectively. Each datapoint represents an independent measure from a single participant. One older participant was excluded as an outlier. (**D**) Recall error rate averaged across recall-demand level (gist and detail) for young (green box; *N* = 34) and older adults (orange box; *N* = 54) was assessed via a linear model. Recall error was higher for older compared to young adults [β = 0.41, *t*(83) = 4.05, *P* < 0.001]. The median is shown as a bold black horizontal line and the 25th and 75th percentiles as black horizontal lines below and above, respectively. Each datapoint represents an independent measure from a single participant. One younger participant was excluded as an outlier, and three older participants were excluded due to methodological problems.

**Table 4:** Accuracy and confidence of young and older adults

	Accuracy		Confidence	
Recognition demand	Young adults (*N* = 35)	Older adults (*N* = 57)	Young adults (*N* = 35)	Older adults (*N* = 57)
Novel foils	0.94 (0.10)	0.90 (0.11)	0.68 (0.25)	0.70 (0.22)
‘Old’ target items	0.74 (0.18)	0.71 (0.17)	0.52 (0.16)	0.51 (0.20)
Easy lure items	0.83 (0.10)	0.64 (0.18)	0.59 (0.18)	0.46 (0.20)
Medium lure items	0.61 (0.15)	0.53 (0.17)	0.49 (0.17)	0.44 (0.21)
Hard lure items	0.48 (0.15)	0.36 (0.17)	0.45 (0.21)	0.46 (0.19)

Mean (standard deviation) for accuracy and confidence of 35 young and 57 older adults. Accuracy is the number of correct trials relative to all trials of the respective recognition demand level. Confidence is the number of trials where participants indicated to be confident in their response relative to all trials of the respective recognition demand level.

We then investigated the effect of recognition-demand level and the interaction of recognition-demand level by age group on recognition confidence (see Table 4 for descriptive values, Fig. 3B and Supplementary Table 3). Here, we additionally assessed a U-shaped relationship in addition to the linear relationship across recognition-demand levels. The model revealed that confidence decreased with increasing recognition-task demand in a linear manner [β = −0.32 (95% CI −0.42, −0.22), *t*(368) = −6.30, *P* < 0.001]. Older adults were less confident than young adults in all recognition-task demand levels [β = −0.43 (95% CI −0.84, −0.01), *t*(92) = −2.03, *P* = 0.044]; however, there was an interaction effect of recognition-task demand (U-shape) by age group [β = 0.24 (95% CI 0.09, 0.39), *t*(368) = 3.11, *P* = 0.002, Cohen’s *f*^2^ = 0.01], with older adults’ confidence showing a U-shape pattern of confidence across demand levels.

Additionally, we investigated the influence of accuracy and the interaction of recognition-demand level by age group by accuracy on recognition confidence (see Table 4 for descriptive values, Supplementary Fig. 1, Supplementary Fig. 2 and Supplementary Table 4). The model revealed that confidence was higher for correct compared to incorrect responses [β = 0.59 (95% CI 0.51, 0.67), *t*(789) = 14.35, *P* < 0.001]. There was an interaction effect of recognition demand by accuracy [β = −0.21 (95% CI −0.29, −0.13), *t*(791) = −5.01, *P* < 0.001, Cohen’s *f*^2^ = 0.10], with confidence for correct responses decreasing while confidence for incorrect responses increasing with higher demand level. Furthermore, there was an interaction effect of age group by accuracy [β = −0.32 (95% CI −0.42, −0.22), *t*(789) = −6.19, *P* < 0.001, Cohen’s *f*^2^ = 0.04], with older adults showing lower confidence compared to young adults for correct responses. The three-way interaction of recognition-demand level by age group by accuracy with older adults showing a descriptively steeper decline in confidence across recognition-task demand compared to young adults for correct responses was not significant [β = −0.10 (95% CI −0.20, −0.00), *t*(790) = −1.91, *P* = 0.056].

Furthermore, we investigated the effect of recall-demand level and the interaction of recall-demand level by age group on recall performance (see Fig. 3C, and Supplementary Table 5) based on a recall scoring system (see Supplementary Methods 2 and Supplementary Table 1). A mean recall score of cued and free recall was used [correlation of cued and free recall: *r* = 0.76 (95% CI 0.64, 0.85), *t*(66) = 9.53, *P* < 0.001]. The model revealed that performance decreased with increasing recall-task demand [β = −0.29 (95% CI −0.34, −0.25), *t*(91) = −13.00, *P* < 0.001]. Older adults recalled less than young adults for both recall-demand levels [β = −1.47 (95% CI −1.83, −1.11), *t*(91) = −8.09, *P* < 0.001]; however, there was an interaction effect of recall-demand level by age group [β = 0.18 (95% CI 0.13, 0.24), *t*(91) = 6.40, *P* < 0.001, Cohen’s *f*^2^ = 0.02], with young adults’ performance showing a steeper decline across demand levels. Caution should be taken regarding this interaction, as the recall score for older adults for gist (mean = 0.028, SD = 0.022) and detail (mean = 0.015, SD = 0.016) is very low compared to young adults’ scores for gist (mean = 0.126, SD = 0.06) and detail (mean = 0.092, SD = 0.053). A score of 0.092 means that 9.2% of the information the participant was exposed to was recalled.

Additionally, we investigated the influence of age group on recall error (see Fig. 3D, and Supplementary Table 6). The model revealed that the recall error was higher for older compared to young adults [β = 0.41 (95% CI 0.21, 0.62), *t*(83) = 4.05, *P* < 0.001], with older adults’ showing on average 2.8 times the error rate of young adults.

Taken together, young adults showed higher recognition accuracy and confidence as well as a higher recall score and lower recall error. Older adults’ accuracy declined more steeply than young adults’s accuracy with increasing recognition-task demand.

### Retrieval performance in older adults related to *APOE4* genotype and Alzheimer’s pathology

Second, only in older adults, we investigated the influence of recognition-demand level, the interaction of recognition-demand level by the *APOE4* group, and the effect of the plasma-based Alzheimer’s pathology on recognition accuracy (see Table 5 for descriptive values, Fig. 4A and Supplementary Table 7). We included age at baseline, sex, vocabulary and working memory capacity in all the following LMMs. The model revealed that accuracy decreased with increasing recognition-task demand (β = −0.70 [95% CI −0.78, −0.61], *t*(260) =−15.65, *P* < 0.001). There was no main effect of the *APOE4* group [β = −0.09 (95% CI −0.28, 0.10), *t*(260) = −0.92, *P* = 0.360] or the AT-Term [β = 0.05 (95% CI −0.03, 0.14), t = 1.16, *P* = 0.245]. However, there was an interaction effect of recognition-demand level by the *APOE4* group [β = −0.18 (95% CI −0.36, −0.01), *t*(260) = −2.06, *P* = 0.041, Cohen’s *f*^2^ = 0.02], with *APOE4* carriers showing a steeper decline in accuracy with higher recognition-task demand. Exploratory post-hoc analysis of the interaction effect with recognition-demand level as a factor revealed a significant difference between age groups for the fifth recognition-demand level (hard lures) [t(295) = 2.465, *P* = 0.0143, SE = 0.049] using emmeans.

**Figure 4:**
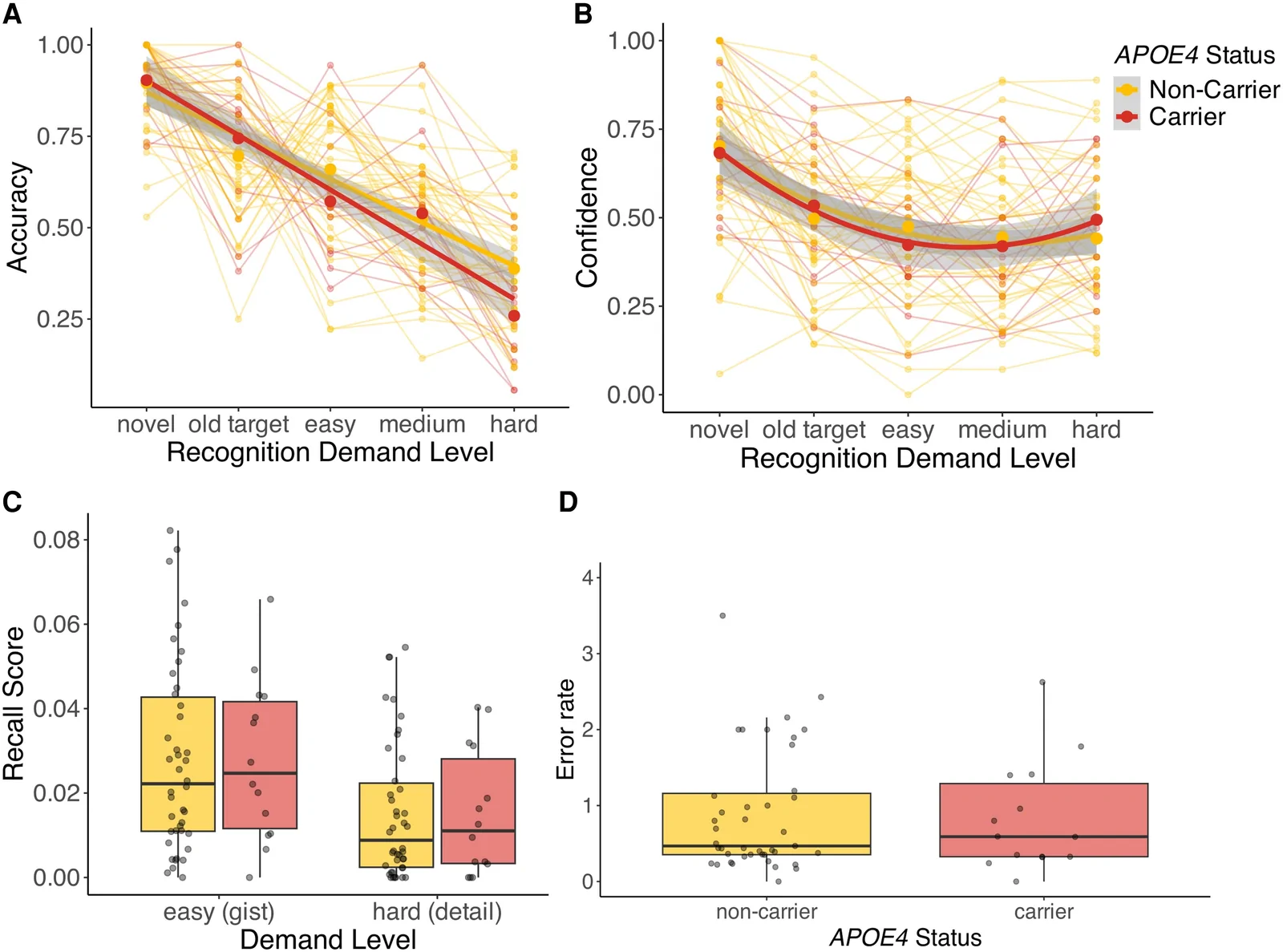
**Recognition-task performance for older *APOE4* non-carriers and carriers.** (**A**) Accuracy across recognition-task demand levels for *APOE4* non-carriers (yellow lines; *N* = 43) and *APOE4* carriers (red lines; *N* = 14) was assessed via a linear mixed model. There was an interaction effect of recognition-demand level by *APOE4* group [β = −0.18, *t*(260) = −2.06, *P* = 0.041]. Presented are group means (bold dots), smoothed group mean trajectories (bold lines) and individual trajectories (fine lines). (**B**) Confidence across recognition-task demand levels for *APOE4* non-carriers (yellow lines; *N* = 43) and *APOE4* carriers (red lines; *N* = 14) was assessed via a linear mixed model. The model revealed that confidence decreased with increasing recognition-task demand in a linear [β = −0.36, t(224) = −8.25, *P* < 0.001] manner, and additionally showed an increase for hard levels, meaning a U-shaped manner [β = 0.25, t(224) = 4.65, *P* < 0.001]. Presented are group means (bold dots), smoothed group mean trajectories (bold lines) and individual trajectories (fine lines). (**C**) Recall score for both recall-demand level (gist and detail) ranging from 0 to 1 for *APOE4* non-carriers (yellow boxes; *N* = 43) and *APOE4* carriers (red boxes; *N* = 13) was assessed via a linear model. Performance decreased with increasing recall-task demand β = −0.32, t(55) = −7.11, *P* < 0.001]. The median is shown as a bold black horizontal line and the 25th and 75th percentiles as black horizontal lines below and above, respectively. Each datapoint represents an independent measure from a single participant. One *APOE4* carrier was excluded as an outlier. (**D**) Recall error rate for both recall-demand level (gist and detail) for *APOE4* non-carriers (yellow boxes; *N* = 41) and *APOE4* carriers (red boxes; *N* = 13) was assessed via a linear model. There was no significant effect (all *P* > 0.05). The median is shown as a bold black horizontal line and the 25th and 75th percentiles as black horizontal lines below and above, respectively. Each datapoint represents an independent measure from a single participant. Three older participants (one *APOE4* carrier and two *APOE4* non-carriers) were excluded due to methodological problems.

**Table 5:** Accuracy and confidence of older *APOE4* carriers and non-carriers

	Accuracy		Confidence	
Recognition demand	Older adults *APOE4* carriers (*N* = 14)	Older adults *APOE4* non-carriers (*N* = 43)	Older adults *APOE4* carriers (*N* = 14)	Older adults *APOE4* non-carriers (*N* = 43)
Novel foils	0.90 (0.09)	0.90 (0.12)	0.68 (0.17)	0.70 (0.23)
‘Old’ target items	0.75 (0.19)	0.70 (0.16)	0.53 (0.19)	0.50 (0.20)
Easy lure items	0.57 (0.17)	0.66 (0.18)	0.42 (0.19)	0.48 (0.21)
Medium lure items	0.54 (0.17)	0.53 (0.17)	0.42 (0.21)	0.44 (0.21)
Hard lure items	0.26 (0.15)	0.39 (0.17)	0.49 (0.17)	0.44 (0.20)

Mean (standard deviation) for accuracy and confidence of older adults (14 *APOE4* carriers, 43 *APOE4* non-carriers separately).

We then investigated the influence of recognition-demand level and the interaction of recognition-demand level by the *APOE4* group on recognition confidence (see Table 5 for descriptive values, Fig. 4B and Supplementary Table 8). Here, we again assessed a U-shaped trajectory in addition to the linear trajectory of confidence across demand levels. The model revealed that confidence decreased with increasing recognition-task demand in a linear [β = −0.36 (95% CI −0.45, −0.28), *t*(224) = −8.25, *P* < 0.001] manner, and additionally showed increase for hard levels, meaning a U-shaped manner [β = 0.25 (95% CI 0.14, 0.35), *t*(224) = 4.65, *P* < 0.001]). There was no significant main effect of the AT-Term or *APOE4* group and no significant interaction with recognition-demand level (all *P* > 0.05, see Supplementary Table 8 for statistics).

Additionally, we investigated the influence of accuracy and the interaction of recognition-demand level by *APOE4* group by accuracy on recognition confidence (see Table 5 for descriptive values, Supplementary Fig. 3, Supplementary Fig. 4 and Supplementary Table 9). The model revealed that confidence was higher for correct compared to incorrect responses [β = 0.29 (95% CI 0.22, 0.36), *t*(486) = 7.70, *P* < 0.001]. There was an interaction effect of recognition demand by accuracy [β = −0.25 (95% CI −0.32, −0.17), *t*(487) = −6.58, *P* < 0.001, Cohen’s *f*^2^ = 0.15], with confidence for correct responses decreasing and confidence for incorrect responses increasing with higher demand level. Furthermore, there was a three-way interaction of recognition-demand level by *APOE4* group by accuracy [β = −0.28 (95% CI −0.42, −0.13), *t*(486) = −3.70, *P* < 0.001, Cohen’s *f*^2^ = 0.02], with *APOE4* carriers compared to non-carriers showing a steeper increase in confidence across recognition-task demand levels for incorrect responses [estimate = −0.11 (95% CI −0.17, −0.04), *t*(494) = −3.27, *p*-FDR = 0.002] and descriptively a steeper decline in confidence across recognition-task demand levels for correct responses [estimate = 0.06 (95% CI −0.02, 0.12), *t*(491) = 1.89, *p*-FDR = 0.060].

VLMT recognition performance was not associated with *APOE4* group or the AT-Term (all *P* > 0.05, see Supplementary Table 10 for statistics), RCFT recognition performance was not associated with *APOE4* group but with the Alzheimer’s pathology ratio, showing worse performance with more pathology [β = −0.47 (95% CI −0.76, −0.18), *t*(50) = −3.30, *P* = 0.002, see Supplementary Table 11 for statistics].

Furthermore, we investigated the effect of recall-demand level and the interaction of recall-demand by *APOE4* group, and the effect of the AT-Term on recall performance (see Fig. 4C and Supplementary Table 12), which was based on a recall scoring system (see Supplementary Methods 2 and Supplementary Table 1). A mean recall score of cued and free recall was used [correlation of cued and free recall: *r* = 0.71 (95% CI 0.55, 0.82), *t*(56) = 7.41, *P* < 0.001]. The model revealed that performance decreased with increasing recall-task demand [β = −0.32 (95% CI −0.41, −0.23), *t*(56) = −7.11, *P* < 0.001]. There was no significant main effect of the AT-Term or *APOE4* group and no significant interaction with recognition demand (all *P* > 0.05, see Supplementary Table 12 for statistics).

Additionally, we investigated the influence of *APOE4* group and the AT-Term on recall error (see Fig. 4D and Supplementary Table 13). There was no significant main effect of the AT-Term or *APOE4* group (all *P* > 0.05, see Supplementary Table 13 for statistics).

Taken together, there were no main effects of *APOE4* carriership or Alzheimer’s pathology in older adults regarding recognition accuracy and confidence, recall score or recall error. In *APOE4* carriers, however, accuracy declined more steeply than in non-carriers with increasing recognition-task demand. *APOE4* carriers also showed a steeper increase in confidence across recognition-task demand levels for incorrect responses compared to non-carriers.

Third, only in older adults, we conducted correlation analysis between EMCOMP recognition accuracy and recall score with established episodic memory scores of the VLMT, RCFT, FCSRT and WMS, as well as with the SDMT for processing speed, the RWT for executive functioning and the CERAD-Plus for general cognition. We used false-discovery-rate corrected *P*-values to account for multiple comparisons.

For recognition accuracy, EMCOMP corrected hit rate was significantly correlated with VLMT corrected hit rate, which, however, did not survive FDR-correction [*r* = 0.26 (95% CI 0.00, 0.49), *t*(56) = 1.99, *p*_FDR_=0.102], but here was no significant correlation with RCFT corrected hit rate [*r* = −0.10 (95% CI −0.35, 0.17), *t*(56) = −0.72, *p*_FDR_ = 0.478]. Mean EMCOMP recall score was significantly positively correlated with all four established episodic memory recall scores (see Fig. 5A–D), namely VLMT [*r* = 0.58 (95% CI 0.37, 0.73), *t*(56) = 5.22, *p*_FDR_ < 0.001], FCSRT [*r* = 0.35 (95% CI 0.10, 0.56), *t*(56) = 2.79, *p*_FDR_ = 0.007], RCFT [*r* = 0.38 (95% CI 0.13, 0.58), *t*(56) = 3.02, *p*_FDR_ = 0.005] and WMS [*r* = 0.44 (95% CI 0.20, 0.63), *t*(56) = 3.60, *p*_FDR_ = 0.001].

**Figure 5:**
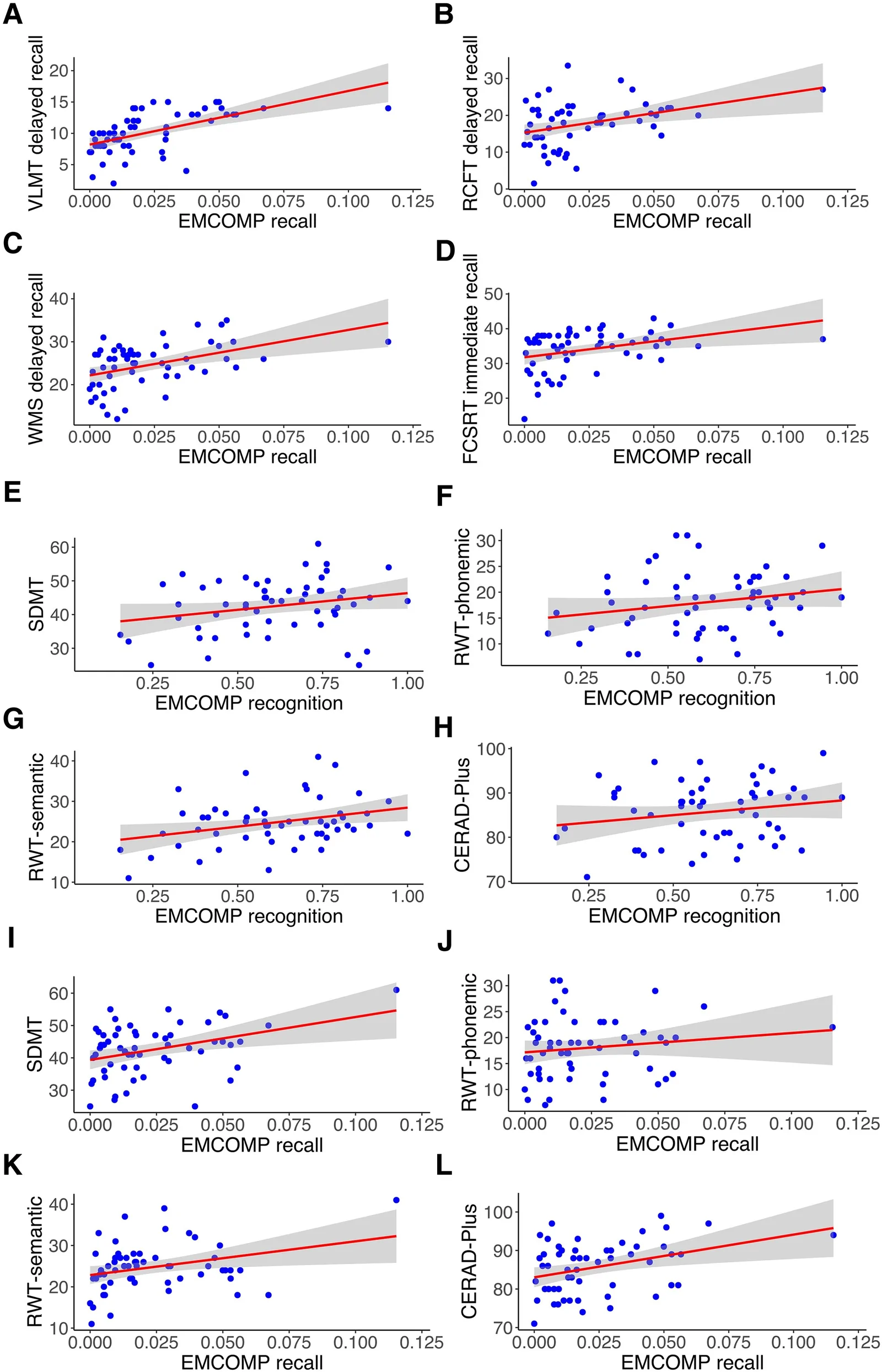
**Pearson correlations of EMCOMP performance and established cognitive tests.** Each datapoint represents an independent measure from a single older participant (*N* = 57). For recall, analyses were repeated when excluding a recall score >0.12. The results remained significant, and the value is not categorized as an outlier, as it is within 2 standard deviations, which was the criterion for outliers. (**A**) Pearson correlation of EMCOMP recall performance and Verbal Learning and Memory Test (VLMT) recall performance (*r* = 0.58, *p*_FDR_ < 0.001). (**B**) Pearson correlation of EMCOMP recall performance and Rey Complex Figure Test and Recognition Trial (RCFT) recall performance (*r* = 0.38, *p*_FDR_ = 0.005). (**C**) Pearson correlation of EMCOMP recall performance and Logical Memory II of the Wechsler Memory Scale (WMS) recall performance (*r* = 0.44, *p*_FDR_ = 0.001). (**D**) Pearson correlation of EMCOMP recall performance and Free and Cued Selective Reminding Test (FCSRT) recall performance (*r* = 0.35, *p*_FDR_ = 0.007). (**E**) Pearson correlation of EMCOMP recognition performance (corrected hit rate) and Symbol Digit Modalities Test (SDMT) performance (*r* = 0.25, *p*_FDR_ = 0.132). (**F**) Pearson correlation of EMCOMP recognition performance (corrected hit rate) and Regensburger Wortflüssigkeitstest (RWT)-phonemic performance (*r* = 0.22, *p*_FDR_ = 0.133). (**G**) Pearson correlation of EMCOMP recognition performance (corrected hit rate) and RWT-semantic performance (*r* = 0.32, *p*_FDR_ = 0.063). (**H**) Pearson correlation of EMCOMP recognition (corrected hit rate) performance and Consortium to Establish a Registry for Alzheimer’s Disease (CERAD)-Plus performance (*r* = 0.20, *p*_FDR_ = 0.156). (**I**) Pearson correlation of EMCOMP recall performance and SDMT performance (*r* = 0.37, *p*_FDR_ = 0.015). (**J**) Pearson correlation of EMCOMP recall performance and RWT-phonemic performance (*r* = 0.14, *p*_FDR_ = 0.301). (**K**) Pearson correlation of EMCOMP recall performance and RWT-semantic performance (*r* = 0.31, *p*_FDR_ = 0.027). (**L**) Pearson correlation of EMCOMP recall performance and CERAD-Plus performance. (*r* = 0.36, *p*_FDR_ = 0.015).

Regarding further cognitive functions, EMCOMP corrected hit rate was not significantly correlated with SDMT, RWT phonemic or semantic fluency, or CERAD-Plus (see Table 6 for statistics and Fig. 5E–H). Mean recall score was significantly positively correlated with SDMT, RWT semantic fluency and CERAD-Plus, but not RWT phonemic fluency (see Table 6 for statistics and Fig. 5I–L).

**Table 6:** Correlation of EMCOMP recognition and recall score with cognitive tests

EMCOMP	Cognitive test	r	95% CI	t	*p*_FDR_
recognition	SDMT	0.25	−0.02, 0.48	1.88	0.132
recognition	RWT phonemic fluency	0.22	−0.04, 0.45	1.67	0.133
recognition	RWT semantic fluency	0.32	0.06, 0.53	2.49	0.063
recognition	CERAD-Plus	0.20	−0.08, 0.44	1.44	0.156
recall score	SDMT	0.37	0.12, 0.57	2.91	0.015
recall score	RWT phonemic fluency	0.14	−0.13, 0.39	1.04	0.301
recall score	RWT semantic fluency	0.31	0.05, 0.53	2.40	0.027
recall score	CERAD-Plus	0.36	0.10, 0.57	2.79	0.015

Correlation *r* and the corresponding 95% confidence interval (CI), *t*-value and false-discovery-rate (FDR) corrected *P*-value. There were 56 degrees of freedom for each correlation model.

## Discussion

### Summary

Our paradigm incorporated multiple levels of retrieval difficulty by systematically varying the required degree of episodic memory specificity. We theorized that increasing specificity requirements would increase task demand. Our approach allowed us to detect early subtle cognitive deficits and to provide novel empirical insights consistent with theories of diminished neural resources with age and Alzheimer’s disease risk. Young adults showed higher recognition accuracy and confidence, a higher recall score and a lower recall error rate compared to older adults. Importantly, we found a steeper decline in recognition accuracy and a steeper increase in high-confidence errors during recognition in older compared to young adults and in older *APOE4* carriers compared to non-carriers with higher retrieval difficulty. There were, however, no associations with early plasma-based Alzheimer’s pathology. Finally, EMCOMP performance was positively correlated with established cognitive tests.

### Retrieval performance in young and older adults

Our first aim was to assess retrieval performance across multiple levels of required memory specificity in young and older cognitively unimpaired adults. In older adults, the recall score declined from gist to detail, and recognition accuracy decreased from novel to ‘old’ target items to easy, medium and hard lure conditions. Young adults showed the same pattern, except that easy lures were rejected more accurately than completely novel items. Our results align with prior studies in young and older adults regarding recall^76,77^ and delayed recognition^58,59^ with different retrieval-demand levels. Notably, particularly the rejection of lures rather than the rejection of novel items and hits for ‘old’ target items was impaired in older adults. This hints towards an increasing cognitive challenge specifically due to the similarity between ‘old’ target items and lures. Item recognition relies on familiarity and gist, whereas lure discrimination depends on recollection of details.^78-80^ The present study extends prior work by a more fine-grained demand manipulation via similarity using three lure levels, offering five recognition-demand levels in total. Additionally, using sentence-based stimuli creates a novel episodic retrieval paradigm for broader application in memory research.

As expected from previous ageing studies,^81-85^ young adults showed higher recognition and recall performance than older adults. Consistent with studies reporting an age-related decline in the ability of mnemonic discrimination and memory specificity,^59,86,87^ older adults already performed around the chance level for medium lures, whereas young adults only did so for hard lures. Furthermore, older adults performed below the chance level for hard lures. Reduced memory specificity may contribute to increased retrieval difficulty in older adults during lure discrimination, particularly when fine-grained episodic detail is required.^88^ Accordingly, our findings may reflect a diminished ability to cope with increasing demands on lure discrimination, in line with the framework proposed by Reuter-Lorenz and Cappell.^39^ Our findings are further consistent with results of a metaanalysis where a larger age difference for recollection-based recognition (discrimination of related and similar items) than for familiarity-based recognition was reported.^89^ Importantly, prior work suggests that while retrieval of specific details declines with age, gist-based retrieval may remain relatively preserved.^90-93^ Thus, a greater reliance on gist-based retrieval could also be an adaptive process to cope with diminished resources and increased demands with age.^93^ For example, Abadie and colleagues conducted a study in which young and older adults studied a list of words that were related and unrelated, representing strong and weak gist relations, respectively. Both age groups successfully retrieved the gist, but older adults struggled in retrieving specific detail, causing increased recognition errors.^90^ Interestingly, a steeper decline in recognition accuracy with older age was not only reported for increasing similarity between items in visual tasks^90^ but also with increasing semantic ambiguity and noise for auditory tasks,^10^ suggesting a modality-independent effect of increasing task demand.

Recognition confidence ratings reflected findings from prior literature.^84,94,95^ Interestingly, while confidence generally decreased with increasing retrieval difficulty, older adults, but not young adults, showed an increase in confidence at the highest difficulty levels, where they gave the most incorrect responses. These findings align with the results from a study by Shing and colleagues^84^ on high-confidence memory errors in an associative recognition paradigm. Even though older adults performed comparably to children when recognizing a word pair, they rated their confidence in errors higher than children did. Age-related differences in memory confidence, particularly the increase of high-confidence errors in older adults, could be attributed to difficulties in binding different components of an episode into a holistic, coherent memory representation.^95^ Due to reduced memory specificity, older adults rely more on the gist of an episode when making memory judgments. Under high recognition demands with high item similarity, this reliance becomes problematic, as difficulties in retrieving specific details hinder lure discrimination and increase the likelihood of high-confidence errors.^94^

Surprisingly, young adults showed a steeper decline in recall performance across increasing retrieval difficulty (from gist to detail) compared to older adults. However, this apparent interaction effect should be interpreted with caution, as older adults’ performance approached floor levels at both manipulation levels. Because their gist-recall performance was already low, further decline at the detail level could not be reliably detected. The paradigm, requiring the verbal recall of numerous information-rich sentences, may have exceeded older adults’ processing capacity. When task demands surpass available resources, cognitive effort decreases and disengagement occurs.^39,40^ Thus, older adults may reach cognitive saturation earlier due to reduced neural resources. These assumptions warrant verification through neuroimaging studies.

### Retrieval performance in older adults related to *APOE4* genotype and Alzheimer’s pathology

Our second aim was to assess the influence of the *APOE4* genotype and plasma-based Alzheimer’s pathology burden on retrieval performance in older adults. We found an effect of *APOE4* on recognition, but not recall performance. Specifically, *APOE4* carriers showed a steeper decline in recognition accuracy and a steeper increase in confidence across difficulty levels for incorrect responses with higher retrieval difficulty. In contrast, no *APOE4*-related effects emerged in the VLMT or RCFT recognition measures, suggesting that the EMCOMP task with multiple levels of difficulty may be more sensitive to subtle, early deficits in retrieval processes.

A study by Rajah and colleagues^50^ employed an intentional spatial context encoding paradigm in middle-aged adults with a family history of sporadic Alzheimer’s disease, the *APOE4* allele and controls. There were two demand levels, as participants either encoded six items (easy) or 12 items (hard). The study found no differences in retrieval accuracy between groups, but differences in activation between groups and demand levels in the hippocampus and parietal cortex. However, there were no interaction effects of group by demand level on activation. Interestingly, participants with Alzheimer’s risk factors exhibited more hippocampal activation during easy encoding trials compared to controls, and higher hippocampal activity during encoding was correlated with better subsequent memory performance. The question remains whether introducing additional levels of task demand, as in our study, would have revealed interaction effects that were not detectable with only two demand levels. A further study^96^ in older adults with mild cognitive impairment reported higher functional connectivity between the hippocampus and parietal cortex in *APOE4* carriers compared to non-carriers, which was positively associated with a memory composite score in the carrier group. While this could hint towards functional compensation, other studies reported the opposite findings.^97^ Overall, the role of *APOE* in ageing and Alzheimer’s disease is multifaceted and remains incompletely understood,^98^ but large cohort studies underline that memory decline is a key feature among cognitively unimpaired *APOE4* carriers^99^ and thus underscore the potential of tasks with varying demand levels for identifying nuanced tipping-points in performance changes.

There were no associations between plasma biomarkers related to Alzheimer’s disease (AT-Term = 1/(Aβ_1–42_/Aβ_1–40_) × p-tau_217_) and EMCOMP recognition or recall performance, nor with VLMT recognition. However, in the RCFT recognition task, a higher plasma-based AT-Term was associated with worse performance. On the one hand, the degree of pathology in this cognitively healthy sample may not yet be sufficient to impact performance or the blood-based markers may not be sensitive enough in this healthy cohort. On the other hand, the absence of behavioural differences despite variability in the AT-Term could be related to functional compensation—that is, comparable behavioural performance despite differences in pathology burden, which could be compensated via specific neural activation or connectivity patterns.^39,49^ In a previous study,^100^ for instance, the authors used a letter Sternberg working memory paradigm with three demand levels (one, three or six letters at the same time). There were no differences in accuracy between Aβ-positive and Aβ-negative cognitively unimpaired older adults, but Aβ-positive individuals showed stronger parametric brain activity increases with higher demand level during the delay period in frontoparietal regions, which was interpreted as compensatory upregulation and additional activation. Another study^101^ reported a parametric increase in brain activity in Aβ-positive cognitively unimpaired older adults during encoding that was positively correlated with episodic memory task performance, specifically the recall of detailed information. Recent findings using multivariate Bayesian decoding in a lifespan sample suggest that age increased the likelihood that higher activation patterns in the cuneal region during problem solving provided non-redundant information.^102^ If higher activation reflects functional compensation as a short-term employment of additional neural resources to meet task demands or if it is part of a pathological cascade of hyperactivation and loss of modulation remains an open question.^45^

Overall, the observed interactions of *APOE4* status and retrieval difficulty on performance may reflect early genotype-related neural resource limitations or alterations in resource allocation via, e.g. decreased network efficiency that precede effects of early pathology with age.^103-106^ Future neuroimaging studies employing the EMCOMP paradigm are needed to address this question.

### Validation of the retrieval task

Our third aim was to assess the relation of the performance on the EMCOMP task and performance on established cognitive tests in older adults. Regarding recognition performance, no significant associations were found between the corrected hit rates for EMCOMP and the widely used memory measures VLMT and RCFT. This could likely reflect, on the one hand, the higher difficulty of the EMCOMP task with additional lure items and, on the other hand, ceiling effects observed in the traditional tests with fewer items and without lures.

For recall, EMCOMP performance showed positive correlations with the established memory recall measures VLMT, FCSRT, RCFT and WMS, indicating convergent validity for retrieval processes. Beyond memory-specific tasks, recall but not recognition performance was positively associated with the SDMT, RWT semantic fluency and the CERAD-Plus composite score. Furthermore, there were positive associations between EMCOMP measures and the covariate working memory capacity, as reported in the Supplementary Tables. These broader associations suggest that successful retrieval in EMCOMP may rely on executive and semantic processes in addition to episodic memory, consistent with higher task demands.

## Limitations

Several limitations of the present study should be noted. First, our cohort was relatively homogeneous in demographic and educational background, which may limit the generalizability of the findings. Additionally, self-reported ancestry cannot substitute for genetically derived ancestry estimates. Second, the sentences used in the EMCOMP task were not fully naturalistic, potentially constraining ecological validity, meaning the generalizability to real-life settings, and are restricted to the German language. Using our method of item construction, we were, however, able to incorporate more social, emotional, perceptual, spatial and temporal details. Furthermore, we assumed that increasing sentence similarity at recognition would lead to a parametric increase in task demand, although this cannot be demonstrated directly within the current design. Third, recall performance in older adults showed evidence of floor effects, indicating that this condition may have been too demanding for this age group and possibly limiting the interpretability of group differences. Additionally, as recognition preceded recall in all participants, exposure to lure items during recognition may have introduced competing memory representations that influenced subsequent recall performance through interference or incorporation of lure content. Fourth, while the current study focused on behavioural performance, no neuroimaging data were yet available. As a next step, we plan to implement the EMCOMP task in the MRI scanner to examine potential functional compensation mechanisms,^36,39^ i.e. preserved behavioural performance despite Alzheimer’s risk or pathology linked to altered activation or connectivity patterns. Additionally, the inclusion of tau-PET imaging, particularly targeting the medial temporal lobe, will allow a closer investigation of the neural correlates of episodic memory integrity and very early Alzheimer’s pathology.^52^ Fifth, the present study is limited by the absence of a full lifespan sample and the restriction of *APOE* genotyping to older adults. Future studies should therefore include middle-aged participants and assess *APOE* status across all age groups to evaluate whether the task is sensitive to early changes emerging prior to late life. Finally, future studies should account for individual differences in cognitive reserve proxies, in addition to functional compensation during the task, which may modulate the relation between pathology, neural activation or connectivity and behavioural performance.

## Conclusion

Our findings indicate that age- and *APOE4*-related differences in episodic memory retrieval with increasing retrieval difficulty could be driven by the rising challenge to meet the higher task demands associated with higher levels of specificity. Vulnerable groups, meaning older adults and *APOE4* carriers, appear less able to overcome these increasing demands, potentially reflecting reduced neural resources. Importantly, the highest demand levels proved too difficult for older adults; as a result, the EMCOMP task effectively spans the full range from manageable to unmanageable challenge. This underscores its suitability for investigating functional compensation with neuroimaging, offering a sensitive testbed for theories of compensatory mechanisms in preclinical stages of cognitive decline.

## Supplementary Material

fcag375_Supplementary_Data

## Data Availability

The datasets generated and/or analysed during the current study are not publicly available due to the inclusion of sensitive participant information and privacy concerns but are available from the corresponding author on reasonable request for replication. Code for statistical analysis is available at https://github.com/fislarissa/EMCOMP_demand.
