# Supplementary material for: Episodic memory retrieval with increasing task demand: associations with age, APOE4 genotype and Alzheimer’s disease pathology
Source: Brain Commun. 2026 Oct 7;8(5):fcag375. doi: 10.1093/braincomms/fcag375 (PMC13690433; doi:10.1093/braincomms/fcag375)
Supplement: Supplementary file 1 [file fcag375_supplementary_data.pdf]

Episodic memory retrieval with increasing task demand: Associations with age, *APOE4* genotype, and Alzheimer's disease pathology

Supplementary Material

Supplementary Methods 1. *APOE* Genotyping

APOE genotyping was available for older participants. Allele frequencies were ε2 = 6.5%, ε3 = 78.5%, and ε4 = 15%. The observed *APOE* genotype distribution was ε3/ε3 = 68%, ε3/ε4 = 16%, ε3/ε2 = 5%, ε4/ε4 = 5%, ε4/ε2 = 4%, and ε2/ε2 = 2%. Hardy–Weinberg equilibrium testing indicated a nominally significant deviation from expected genotype frequencies (χ^2 = 9.06, p = .037). However, several considerations suggest that the observed deviation revealed by Hardy–Weinberg equilibrium testing is unlikely to fully account for the reported effects. First, the deviation is based on a relatively small sample size (N = 57), in which genotype counts are strongly influenced by a small number of individuals. Second, the observed *APOE* genotype distribution is comparable to frequencies reported in large European ancestry cohorts (e.g., Farrer et al., 1997). Third, additional adjustment for *APOE2* carrier status did not change the results in any of the models below, suggesting that the findings are not driven by the distribution of ε2 alleles. As an example, we provide the results of the model examining the effect of recognition-demand level on accuracy (Supplementary Table S7) including *APOE2* status (highlighted in green):

Linear model of effects of recognition-demand level on accuracy in older adults including *APOE2* status

	Accuracy							
Predictors	*Estimates*	*std. Error*	*std. Beta*	*standardized std. Error*	*CI*	*standardized CI*	*Statistic*	*p*
(Intercept)	0.57	0.13	0.07	0.06	0.32 – 0.82	-0.05 – 0.19	4.50	**<0.001**
Recognition-demand level	-0.17	0.01	-0.70	0.04	-0.19 – -0.15	-0.78 – -0.61	-15.67	**<0.001**
APOE4 Group [Carrier]	-0.02	0.02	-0.09	0.10	-0.07 – 0.02	-0.29 – 0.10	-0.97	0.332
Alzheimer's pathology	0.01	0.01	0.06	0.04	-0.01 – 0.04	-0.03 – 0.15	1.36	0.174
Age at baseline	0.05	0.03	0.06	0.04	-0.01 – 0.11	-0.01 – 0.14	1.60	0.111
Sex [Male]	-0.02	0.02	-0.10	0.08	-0.06 – 0.01	-0.26 – 0.06	-1.26	0.207

Vocab score	0.00	0.00	0.02	0.04	-0.00 – 0.00	-0.07 – 0.10	0.35	0.725
Working memory score	0.03	0.01	0.14	0.04	0.01 – 0.05	0.06 – 0.23	3.37	**0.001**
APOE2 Group [Carrier]	-0.03	0.03	-0.04	0.04	-0.09 – 0.03	-0.12 – 0.04	-0.91	0.361
Recognition-demand level × *APOE4* Group [Carrier]	-0.04	0.02	-0.18	0.09	-0.09 – -0.00	-0.36 – -0.01	-2.06	**0.040**
**Random Effects**								
σ^2	0.02							
$\tau_{00\ participant}$	0.00							
$N_{participant}$	56							
Observations	280							
Marginal R^2 / Conditional R^2	0.588 / NA							

Model: *Accuracy ~ Recognition-demand level (linear) × APOE4 group + Alzheimer's pathology (AT-Term) + Age at baseline + Sex + Vocabulary + Working memory capacity*, with random intercept for participant. N participant = 57 (14 *APOE4* carriers, 43 non-carriers). Estimates, standardized betas, 95% confidence intervals, and test statistics are shown for each predictor.

The interaction between recognition-demand level and *APOE4* carrier status remained significant (β = −0.04, *p* = .040), while *APOE2* carrier status was not significantly associated with accuracy (β = −0.03, *p* = .361) (highlighted in green).

Supplementary Methods 2. Recall scoring system

To evaluate the recall session, a scoring system was adapted based on prior research that distinguished between gist and detail information (Bird et al., 2015; Burke et al., 1992; Wessel et al., 2015). The scoring system separates the components of the sentences in two main categories: central (= gist sum score) and peripheral information (= detail sum score). The two scores represent the two recall demand levels. The gist sum score includes information about social relations (SOC - social central) and action (AC - action central) in the sentence. The

detail sum score consists of five subcategories: social (SOP - social peripheral), emotional (EP - emotional peripheral), perceptual (PP - perceptual peripheral), spatial (SP - spatial peripheral) and temporal (TP - temporal peripheral) information. Each of the 75 encoded sentences was scored using the scoring system to predefine the maximum number of points possible for each category and the total sum scores, respectively. Then the free (90 minutes) and cued recall (100 minutes) were scored by giving one point for a correctly recalled piece of information in the respective category. Half a point was given when a word was recalled with similar content to the encoded (correct) word, e.g. saying "holiday" instead of "vacation". The total scores for each category were collected, as well as a total sum score for gist (total gist = SC + AC) and detail (total detail = SOP + EP + PP + SP + TP). The scoring also accounts for errors, including swaps and add ons. Swaps mean switching information from different encoded sentences; and add ons mean additional information that was not encoded. Two examples for a free recall scoring can be found in Table S1.

After scoring each recall type, the total scores were transformed into relative scores, meaning they were relative to the maximum possible score in the free or cued recall, respectively. Finally, a total recall score including information from both cued and free recall for either gist or detail information was built (free recall detail + cued recall detail; free recall gist cued recall gist). The relative total error score included swaps and add ons in cued and free recall in relation to the total recall score. Therefore, the relative error scores rather represent an error rate. If the error rate is greater than 1, it means the person recalled more information correctly than they made errors. If the rate equals 1, the person made as many errors as correct recalls and if the rate is smaller than 1 it indicates more errors than correct recalls.

Supplementary Table 1. Examples for the recall scoring

	**Example 1**						
Example 1	**Marvin** in **dunkelblauer Hose**, den sein **Vater** **unterstützt**, wird im **Sommerurlaub** **reich**.<br>*Marvin in dark blue pants, whom his father supports, is getting rich on summer vacation.*						
Scoring categories	**SOC**	**SOP**	**AC**	**EP**	**PP**	**SP**	**TP**
Maximum score	2	-	1	1	2	-	1
	**Free recall 1**						
Recall example 1	„**Jemand** wird **reich**…**unterstützt** von seinem **Vater** während der **Sommerferien**"<br>*"Someone is getting rich… supported by his father during the summer holidays."*						

Scoring categories	**SOC**	**SOP**	**AC**	**EP**	**PP**	**SP**	**TP**	**swaps**	**add on**
Scoring	1,5	-	1	1	0	-	0,5	-	-

	**Example 2**								
Example 2	**Oma Katharina** in ihrem **grünen** **Haus**, die ihre **Enkel** in den **Ferien** **verwöhnt**, ist **fürsorglich**. *"Grandma Katharina in her green house, who is pampering her grandchildren during the summer holidays, is caring."*								
Scoring categories	**SOC**	**SOP**	**AC**	**EP**	**PP**	**SP**	**TP**		
Maximum score	2	-	1	1	2	-	1		
	**Free recall 2**								
Recall example 2	„**Oma Katharina** hat ihre **Enkel** bei den **Hausaufgaben** **unterstützt…im** **grünen** **Haus**" *"Grandma Katharina supported her grandchildren with their homework…in a green garden house."*								
Scoring categories	**SOC**	**SOP**	**AC**	**EP**	**PP**	**SP**	**TP**	**swaps**	**add on**
Scoring	2	1	0	0	1	1	0	1	1

Demonstration of scoring two sentences with the recall scoring system, showing the maximum scores possible and an example scoring for a free recall response in German with a literal translation in English below. The scoring system accounts for correctly recalled gist (central) information, details (peripheral) and recall errors (swaps and add ons). Abbreviations: SOC…social central, SOP…social peripheral, AC…action central, EP…emotional peripheral, PP…perceptual peripheral, SP…spatial peripheral, TP…temporal peripheral.

Supplementary Table 2. Linear model of effects of recognition-demand level on accuracy in young and older adults

	Accuracy							
Predictors	*Estimates*	*std. Error*	*std. Beta*	*standardized std. Error*	*CI*	*standardized CI*	*Statistic*	*p*
(Intercept)	0.73	0.10	0.27	0.07	0.54 – 0.92	0.13 – 0.41	7.51	**<0.001**
Recognition-demand level	-0.15	0.01	-0.63	0.05	-0.17 – -0.13	-0.73 – -0.53	-12.71	**<0.001**
Age group [Older adults]	-0.08	0.02	-0.35	0.09	-0.12 – -0.04	-0.52 – -0.18	-3.99	**<0.001**

Sex [Male]	-0.03	0.01	-0.12	0.06	-0.06 – 0.00	-0.24 – 0.01	-1.86	0.063
Vocab score	-0.00	0.00	-0.00	0.04	-0.00 – 0.00	-0.08 – 0.08	-0.04	0.970
Working memory score	0.03	0.01	0.13	0.03	0.02 – 0.05	0.06 – 0.19	3.87	**<0.001**
Recognition-demand level × Age group [Older adults]	-0.03	0.01	-0.13	0.06	-0.06 – -0.00	-0.25 – -0.00	-2.01	**0.045**
**Random Effects**								
σ^2	0.02							
$\tau_{00\ participant}$	0.00							
$N_{participant}$	92							
Observations	460							
Marginal R^2 / Conditional R^2	0.567 / NA							

Model: *Accuracy ~ Recognition-demand level (linear) × Age group + Sex + Vocabulary + Working memory capacity*, with random intercept for participant. N participant = 92 (35 young, 57 older adults). Estimates, standardized betas, 95% confidence intervals, and test statistics are shown for each predictor. Exploratory post-hoc analysis of the interaction effect with recognition-demand level as a factor revealed a significant difference between age groups for the third recognition-demand level (easy lures) (t(434) = 5.179, $p < 0.001$, SE = 0.035) and the fifth recognition-demand level (hard lures) (t(224) = 3.242, $p = 0.001$, SE = 0.035) using emmeans.

Supplementary Table 3. Linear model of effects of recognition-demand level on confidence in young and older adults

				Confidence				
Predictors	*Estimates*	*std. Error*	*std. Beta*	*standardized std. Error*	*CI*	*standardized CI*	*Statistic*	*p*
(Intercept)	0.44	0.20	0.23	0.17	0.03 – 0.84	-0.11 – 0.57	2.15	**0.034**
Recognition-demand level	-0.07	0.01	-0.32	0.05	-0.09 – -0.05	-0.42 – -0.22	-6.30	**<0.001**

Age group [Older adults]	-0.09	0.05	-0.43	0.21	-0.19 – -0.00	-0.84 – -0.01	-2.03	**0.044**
Recognition-demand level U-shape	0.01	0.01	0.04	0.06	-0.02 – 0.04	-0.07 – 0.16	0.74	0.460
Sex [Male]	-0.07	0.03	-0.33	0.14	-0.13 – -0.01	-0.61 – -0.06	-2.39	**0.019**
Vocab score	0.00	0.00	0.06	0.09	-0.00 – 0.01	-0.13 – 0.24	0.63	0.532
Working memory score	0.03	0.02	0.13	0.07	-0.00 – 0.06	-0.02 – 0.28	1.74	0.086
Recognition-demand level × Age group [Older adults]	-0.01	0.01	-0.04	0.06	-0.04 – 0.02	-0.17 – 0.09	-0.61	0.540
Recognition-demand level U-shape × Age group [Older adults]	0.05	0.02	0.24	0.08	0.02 – 0.09	0.09 – 0.39	3.11	**0.002**
**Random Effects**								
σ^2	0.02							
$\tau_{00\ participant}$	0.02							
ICC	0.44							
$N_{participant}$	92							
Observations	460							
Marginal R^2 / Conditional R^2	0.206 / 0.552							

Model: *Confidence ~ Recognition-demand level (linear) × Age group + Recognition-demand level (U-shape) × Age group + Sex + Vocabulary + Working memory capacity*, with random intercept for participant. N participant = 92 (35 young, 57 older adults). Estimates, standardized betas, 95% confidence intervals, and test statistics are shown for each predictor.

Supplementary Table 4. Linear model of effects of recognition-demand level and accuracy on confidence in young and older adults

	Confidence									
Predictors	*Estimates*	*std. Error*	*std. Beta*	*standardized std. Error*	*CI*	*standardized CI*	*Statistic*	*std. Statistic*	*p*	*std. p*

(Intercept)	0.23	0.21	0.05	0.12	-0.18 – 0.64	-0.19 – 0.29	1.13	0.43	0.261	0.669
Recognition-demand level	0.07	0.02	0.02	0.04	0.03 – 0.11	-0.07 – 0.10	3.71	0.38	**<0.001**	0.702
Age group [Older adults]	0.11	0.05	0.03	0.15	0.02 – 0.20	-0.26 – 0.32	2.31	0.18	**0.022**	0.860
Accuracy	0.36	0.02	0.59	0.04	0.31 – 0.41	0.51 – 0.67	14.59	14.35	**<0.001**	**<0.001**
Sex [Male]	-0.06	0.03	-0.18	0.10	-0.12 – 0.01	-0.39 – 0.02	-1.79	-1.79	0.077	0.077
Vocab score	0.00	0.00	0.01	0.07	-0.00 – 0.00	-0.13 – 0.15	0.17	0.17	0.864	0.864
Working memory score	0.02	0.02	0.07	0.06	-0.01 – 0.05	-0.04 – 0.18	1.19	1.19	0.236	0.236
Recognition-demand level × Age group [Older adults]	0.00	0.02	-0.10	0.05	-0.05 – 0.05	-0.20 – 0.00	0.07	-1.90	0.941	0.058
Recognition-demand level × Accuracy	-0.13	0.03	-0.21	0.04	-0.18 – -0.08	-0.29 – -0.13	-5.01	-5.01	**<0.001**	**<0.001**
Age group [Older adults] × Accuracy	-0.19	0.03	-0.32	0.05	-0.25 – -0.13	-0.42 – -0.22	-6.04	-6.19	**<0.001**	**<0.001**
Recognition-demand level × Age group [Older adults] × Accuracy	-0.06	0.03	-0.10	0.05	-0.12 – 0.00	-0.20 – 0.00	-1.91	-1.91	0.056	0.056
**Random Effects**										
σ^2	0.05									
$\tau_{00\ participant}$	0.02									
ICC	0.25									
$N_{participant}$	92									
Observations	879									
Marginal R^2 / Conditional R^2	0.270 / 0.454									

Model: *Confidence ~ Recognition-demand level (linear) × Age group × Accuracy + Sex + Vocabulary + Working memory capacity*, with random intercept for participant. N participant = 92 (35 young, 57 older adults). Estimates, standardized betas, 95% confidence intervals, and test statistics are shown for each predictor.

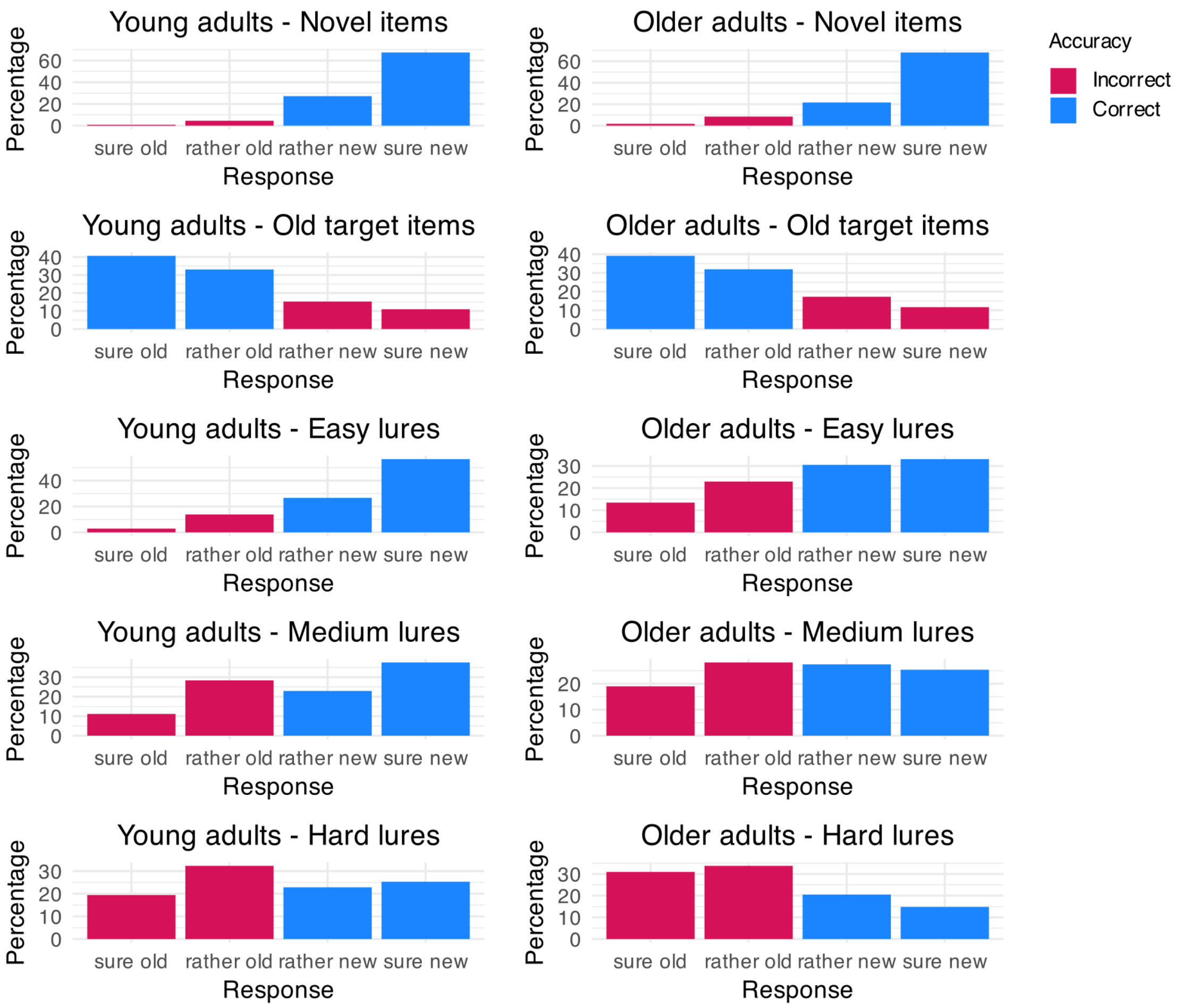

Supplementary Figure 1. Distribution of confidence ratings and their accuracy in young and older adults. Proportion of trials assigned to each confidence category split by response accuracy (correct (in blue) versus incorrect (in red)), for young (N=35) and older adults (N=57) across the five recognition-demand levels. Each bar represents the group-level proportion of trials per confidence category and accuracy status. Underlying statistical model: linear mixed-effects model of confidence as a function of recognition-demand level, age group, and accuracy (see Supplementary Table S4; *Confidence ~ Recognition-demand level (linear) × Age group × Accuracy + Sex + Vocabulary + Working memory capacity,* random intercept for participant).

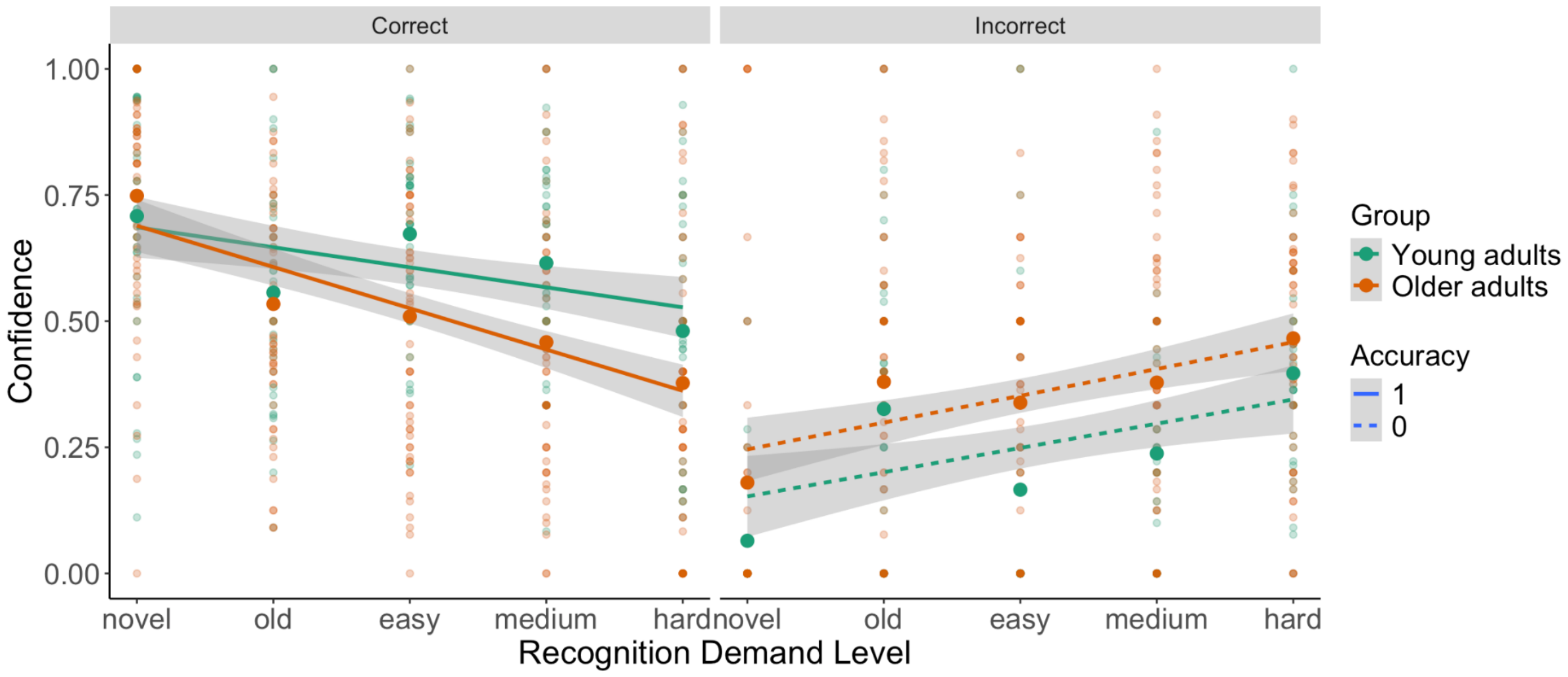

Supplementary Figure 2. Confidence by recognition-demand level, age group, and accuracy. Confidence ratings across the five recognition-demand levels (novel, old, easy, medium, hard lures), separately for correct (left panel) and incorrect (right panel) responses, in young (green; N = 35) and older adults (orange; N = 57). Small, semi-transparent points represent individual trial-level confidence values; large, opaque points represent group means at each recognition-demand level. Lines show unadjusted linear regression fits per group and accuracy condition (solid = correct, dashed = incorrect), with shaded areas representing 95% confidence intervals; these fits are not adjusted for covariates and are shown for visualization only — statistical inference is based on the covariate-adjusted linear mixed-effects model reported in Supplementary Table S4 (*Confidence ~ Recognition-demand level (linear) × Age group × Accuracy + Sex + Vocabulary + Working memory capacity*, random intercept for participant): interaction of recognition-demand level × accuracy (β = -0.21, t(791) = -5.01, p < 0.001); interaction of age group × accuracy (β = -0.32, t(789) = -6.19, p < 0.001); three-way interaction of recognition-demand level × age group × accuracy (β = -0.10, t(790) = -1.91, p = 0.056).

Supplementary Table 5. Linear model of effects of recall-demand level on recall score in young and older adults

	Recall score							
Predictors	*Estimates*	*std. Error*	*std. Beta*	*standardized std. Error*	*CI*	*standardized CI*	*Statistic*	*p*
(Intercept)	0.13	0.05	1.07	0.15	0.03 – 0.23	0.78 – 1.37	2.60	**0.011**
Recall-demand level	-0.02	0.00	-0.29	0.02	-0.02 – -0.01	-0.34 – -0.25	-13.00	**<0.001**
Age group [Older adults]	-0.09	0.01	-1.47	0.18	-0.11 – -0.06	-1.83 – -1.11	-8.09	**<0.001**
Sex [Male]	-0.02	0.01	-0.35	0.13	-0.04 – -0.01	-0.60 – -0.09	-2.69	**0.008**

Vocab score	-0.00	0.00	-0.02	0.09	-0.00 – 0.00	-0.19 – 0.15	-0.23	0.818
Working memory score	0.00	0.00	0.05	0.07	-0.01 – 0.01	-0.09 – 0.18	0.65	0.520
Recall-demand level × Age group [Older adults]	0.01	0.00	0.18	0.03	0.01 – 0.01	0.13 – 0.24	6.40	**<0.001**
**Random Effects**								
σ^2	0.00							
$\tau_{00\ participant}$	0.00							
ICC	0.91							
$N_{participant}$	91							
Observations	182							
Marginal R^2 / Conditional R^2	0.608 / 0.965							

Model: *Recall score ~ Recall-demand level (linear) × Age group + Sex + Vocabulary + Working memory capacity*. N participant = 91. Estimates, standardized betas, 95% confidence intervals, and test statistics are shown for each predictor. One older participant was excluded as an outlier with values > 2 standard deviations from the mean for older adults (gist recall score > 0.083 and detail recall score > 0.054).

Supplementary Table 6. Linear model of effects of age group on recall error score in young and older adults

	**Recall error score (log transformed)**											
Predictors	*Estimates*	*std. Error*	*std. Beta*	*exp Beta*	*% change*	*standardized std. Error*	*CI*	*standardized CI*	*Statistic*	*std. Statistic*	*p*	*std. p*
(Intercept)	-2.11	1.05	0.25	0.122	-87.80	0.08	-4.20 – -0.02	0.09 – 0.42	-2.01	3.07	**0.048**	**0.003**
Age group [Older adults]	1.02	0.22	0.41	2.76	176.00	0.10	0.57 – 1.46	0.21 – 0.62	4.57	4.05	**<0.001**	**<0.001**
Sex [Male]	0.12	0.16	0.08	1.13	13.00	0.07	-0.20 – 0.44	-0.07 – 0.22	0.76	1.08	0.448	0.285
Vocab score	0.01	0.01	-0.00	1.01	0.50	0.05	-0.02 – 0.03	-0.10 – 0.09	0.47	-0.07	0.638	0.944

Working memory score	-0.29	0.09	-0.12	0.75	-20.00	0.04	-0.47 – -0.11	-0.20 – -0.05	-3.14	-3.17	**0.002**	**0.002**
Observations	88											
R^2 / R^2 adjusted	0.440 / 0.413											

Model: *Recall error rate ~ Age group + Sex + Vocabulary + Working memory capacity.* Estimates, standardized betas, 95% confidence intervals, and test statistics are shown for each predictor. Regression coefficients (β) are additionally back-transformed via exponentiation (exp(β)) with corresponding percent (%) change, for interpretation on the original scale. Three older individuals were excluded for this analysis, as there were methodological problems with their recall error scoring. One younger participant was excluded as an outlier with values > 2 standard deviations from the mean for younger adults (error rate > 0.9).

Supplementary Table 7. Linear model of effects of recognition-demand level on accuracy in older adults

	Accuracy							
Predictors	*Estimates*	*std. Error*	*std. Beta*	*standardized std. Error*	*CI*	*standardized CI*	*Statistic*	*p*
(Intercept)	0.57	0.13	0.07	0.06	0.32 – 0.82	-0.04 – 0.19	4.51	**<0.001**
Recognition-demand level	-0.17	0.01	-0.70	0.04	-0.19 – -0.15	-0.78 – -0.61	-15.65	**<0.001**
APOE4 Group [Carrier]	-0.02	0.02	-0.09	0.10	-0.07 – 0.02	-0.28 – 0.10	-0.92	0.360
Alzheimer's pathology	0.01	0.01	0.05	0.04	-0.01 – 0.03	-0.03 – 0.14	1.16	0.245
Age at baseline	0.05	0.03	0.06	0.04	-0.01 – 0.12	-0.01 – 0.14	1.65	0.099
Sex [Male]	-0.03	0.02	-0.11	0.08	-0.06 – 0.01	-0.26 – 0.05	-1.37	0.172
Vocab score	0.00	0.00	0.01	0.04	-0.00 – 0.00	-0.07 – 0.10	0.31	0.756

Working memory score	0.03	0.01	0.15	0.04	0.01 – 0.05	0.06 – 0.23	3.48	**0.001**
Recognition-demand level × *APOE4* Group [Carrier]	-0.04	0.02	-0.18	0.09	-0.09 – -0.00	-0.36 – -0.01	-2.06	**0.041**
**Random Effects**								
σ^2	0.02							
$\tau_{00\ participant}$	0.00							
$N_{participant}$	57							
Observations	280							
Marginal R^2 / Conditional R^2	0.586 / NA							

Model: *Accuracy ~ Recognition-demand level (linear) × APOE4 group + Alzheimer's pathology (AT-Term) + Age at baseline + Sex + Vocabulary + Working memory capacity*, with random intercept for participant. N participant = 57 (14 *APOE4* carriers, 43 non-carriers). Estimates, standardized betas, 95% confidence intervals, and test statistics are shown for each predictor. Exploratory post-hoc analysis of the interaction effect with recognition-demand level as a factor revealed a significant difference between age groups for the fifth recognition-demand level (hard lures) ($t(295) = 2.465$, $p = 0.0143$, $SE = 0.049$) using emmeans.

Supplementary Table 8. Linear model of effects of recognition-demand level on confidence in older adults

	Confidence							
Predictors	*Estimates*	*std. Error*	*std. Beta*	*standardized std. Error*	*CI*	*standardized CI*	*Statistic*	*p*
(Intercept)	0.26	0.27	-0.06	0.14	-0.27 – 0.80	-0.33 – 0.22	0.99	0.324
Recognition-demand level	-0.08	0.01	-0.36	0.04	-0.10 – -0.06	-0.45 – -0.28	-8.25	**<0.001**
APOE4 Group [Carrier]	0.00	0.05	0.01	0.24	-0.10 – 0.11	-0.46 – 0.49	0.05	0.957
Recognition-demand level U-shape	0.05	0.01	0.25	0.05	0.03 – 0.08	0.14 – 0.35	4.65	**<0.001**
Alzheimer's pathology	-0.01	0.02	-0.04	0.10	-0.05 – 0.03	-0.23 – 0.15	-0.43	0.671

Age at baseline	-0.02	0.06	-0.03	0.09	-0.15 – 0.11	-0.20 – 0.15	-0.31	0.761
Sex [Male]	-0.11	0.04	-0.47	0.17	-0.18 – -0.03	-0.82 – -0.13	-2.74	**0.008**
Vocab score	0.00	0.00	0.10	0.10	-0.00 – 0.01	-0.09 – 0.29	1.06	0.296
Working memory score	0.02	0.02	0.10	0.09	-0.02 – 0.06	-0.09 – 0.29	1.06	0.296
Recognition-demand level × *APOE4* Group [Carrier]	0.01	0.02	0.05	0.09	-0.03 – 0.05	-0.12 – 0.23	0.58	0.560
Recognition-demand level U-shape × *APOE4* Group [Carrier]	0.02	0.02	0.11	0.11	-0.02 – 0.07	-0.10 – 0.32	1.04	0.298
**Random Effects**								
σ^2	0.02							
$\tau_{00\ participant}$	0.02							
ICC	0.44							
$N_{participant}$	57							
Observations	280							
Marginal R^2 / Conditional R^2	0.268 / 0.591							

Model*: Confidence ~ Recognition-demand level (linear) × APOE4 group + Recognition-demand level (U-shape) × APOE4 group + Alzheimer's pathology (AT-Term) + Age at baseline + Sex + Vocabulary + Working memory capacity,* with random intercept for participant. N participant = 57. Estimates, standardized betas, 95% confidence intervals, and test statistics are shown for each predictor.

Supplementary Table 9. Linear model of effects of recognition-demand level and accuracy on confidence in older adults

	Confidence									
Predictors	*Estimates*	*std. Error*	*std. Beta*	*standardized std. Error*	*CI*	*standardized CI*	*Statistic*	*std. Statistic*	*p*	*std. p*
(Intercept)	0.28	0.27	0.17	0.10	-0.26 – 0.81	-0.04 – 0.37	1.03	1.64	0.307	0.107
Recognition-demand level	0.05	0.02	-0.10	0.04	0.01 – 0.08	-0.18 – -0.03	2.79	-2.71	**0.005**	**0.007**

APOE4 Group [Carrier]	-0.00	0.05	-0.08	0.17	-0.11 – 0.11	-0.41 – 0.26	-0.06	-0.45	0.949	0.657
Accuracy	0.18	0.02	0.29	0.04	0.13 – 0.22	0.22 – 0.36	8.00	7.70	**<0.001**	**<0.001**
Alzheimer's pathology	-0.00	0.02	-0.01	0.08	-0.05 – 0.04	-0.16 – 0.15	-0.07	-0.07	0.944	0.944
Age at baseline	-0.01	0.07	-0.01	0.07	-0.14 – 0.13	-0.14 – 0.13	-0.10	-0.10	0.919	0.919
Sex [Male]	-0.10	0.04	-0.35	0.14	-0.18 – -0.02	-0.62 – -0.07	-2.55	-2.55	**0.014**	**0.014**
Vocab score	0.00	0.00	0.04	0.07	-0.00 – 0.01	-0.11 – 0.19	0.52	0.52	0.605	0.605
Working memory score	0.01	0.02	0.05	0.07	-0.03 – 0.05	-0.10 – 0.20	0.65	0.65	0.520	0.520
Recognition-demand level × *APOE4* Group [Carrier]	0.11	0.03	0.07	0.07	0.04 – 0.17	-0.07 – 0.22	3.29	0.97	**0.001**	0.335
Recognition-demand level × Accuracy	-0.15	0.02	-0.25	0.04	-0.19 – -0.11	-0.32 – -0.17	-6.58	-6.58	**<0.001**	**<0.001**
APOE4 Group [Carrier] × Accuracy	-0.04	0.04	-0.08	0.07	-0.13 – 0.05	-0.22 – 0.07	-0.86	-1.04	0.391	0.301
Recognition-demand level × *APOE4* Group [Carrier] × Accuracy	-0.17	0.05	-0.28	0.07	-0.25 – -0.08	-0.42 – -0.13	-3.70	-3.70	**<0.001**	**<0.001**
**Random Effects**										
σ^2	0.05									
$\tau_{00\ participant}$	0.02									
ICC	0.25									
$N_{participant}$	57									
Observations	541									
Marginal R^2 / Conditional R^2	0.244 / 0.431									

Model: *Confidence ~ Recognition-demand level (linear) × APOE4 group × Accuracy + Alzheimer's pathology (AT-Term) + Age at baseline + Sex + Vocabulary + Working memory capacity*, with random intercept for

participant. N participant = 57. Estimates, standardized betas, 95% confidence intervals, and test statistics are shown for each predictor.

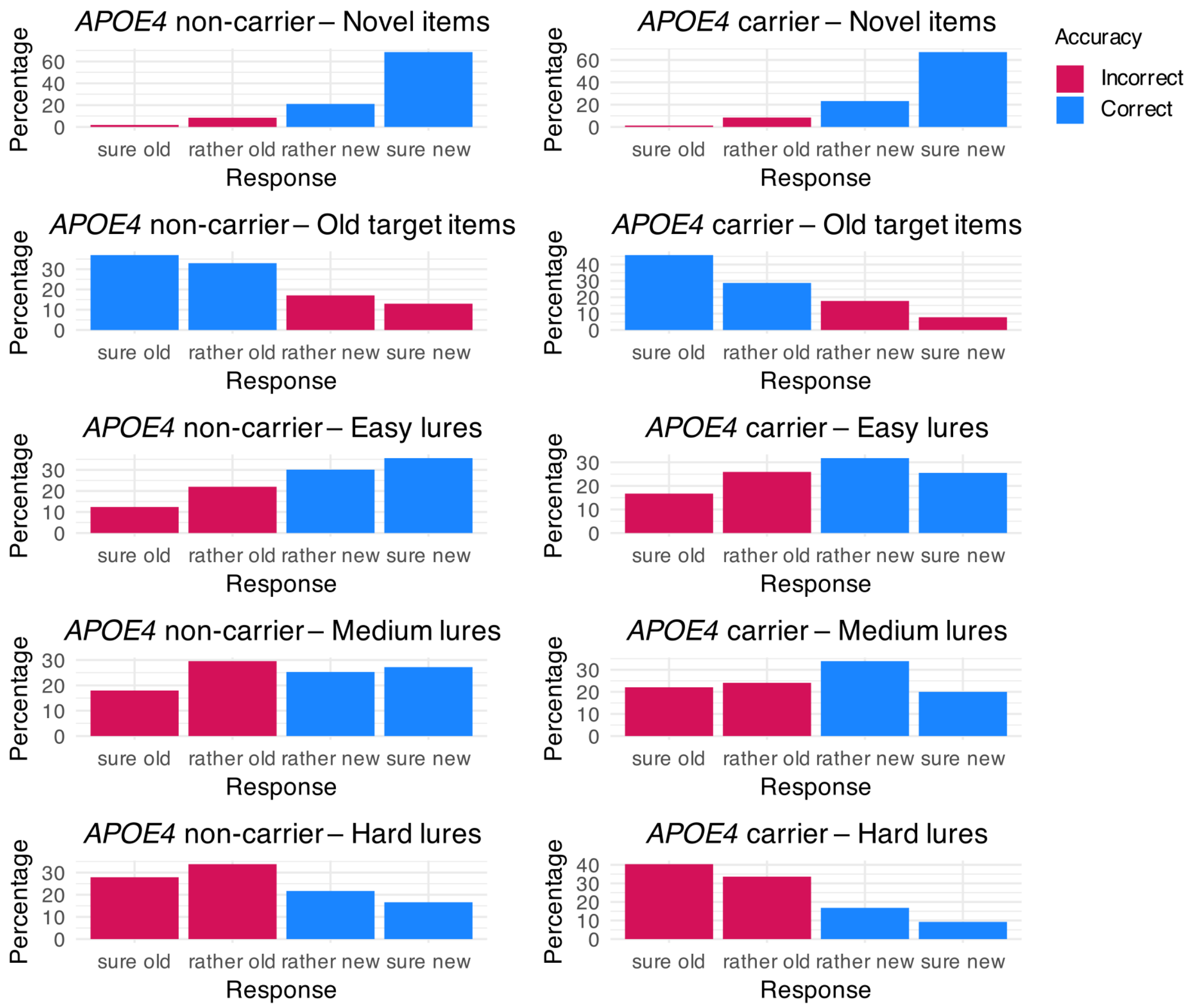

Supplementary Figure 3. Distribution of confidence ratings and their accuracy in older *APOE4* carriers and non-carriers. Proportion of trials assigned to each confidence category split by response accuracy (correct (in blue) versus incorrect (in red)), for older *APOE4* carriers (N=14) and non-carriers (N=43) across the five recognition-demand levels. Each bar represents the group-level proportion of trials per confidence category and accuracy status. Underlying statistical model: linear mixed-effects model of confidence as a function of recognition-demand level, age group, and accuracy (see Supplementary Table S9; *Confidence ~ Recognition-demand level (linear) × APOE4 group × Accuracy + Alzheimer's pathology + Age + Sex + Vocabulary + Working memory capacity*, random intercept for participant).

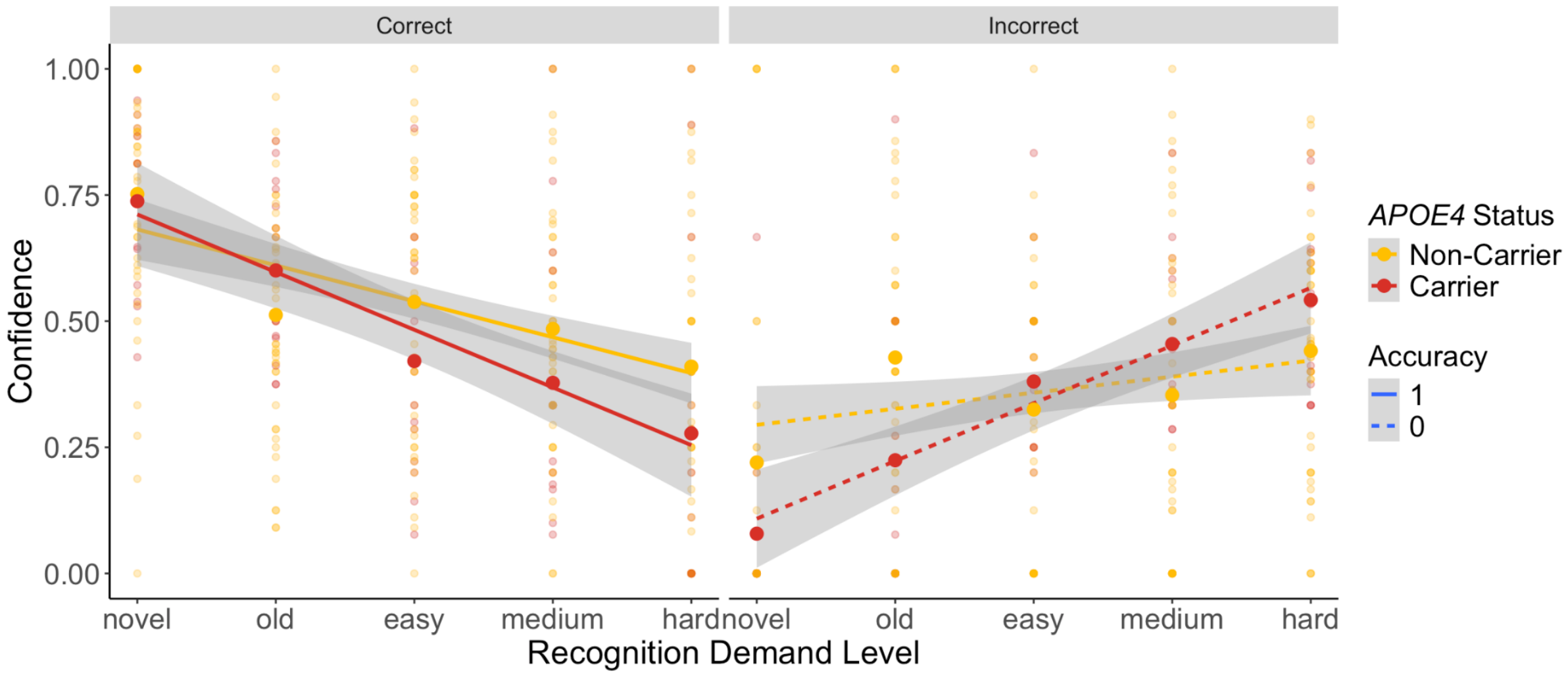

Supplementary Figure 4. Confidence by recognition-demand level, *APOE4* group, and accuracy. Confidence ratings across the five recognition-demand levels (novel, old, easy, medium, hard lures), separately for correct (left panel) and incorrect (right panel) responses, in older *APOE4* non-carriers (yellow; N = 43) and carriers (red; N = 14). Small, semi-transparent points represent individual trial-level confidence values; large, opaque points represent group means at each recognition-demand level. Lines show unadjusted linear regression fits per group and accuracy condition (solid = correct, dashed = incorrect), with shaded areas representing 95% confidence intervals; these fits are not adjusted for covariates and are shown for visualization only — statistical inference is based on the covariate-adjusted linear mixed-effects model reported in Supplementary Table S9 (*Confidence ~ Recognition-demand level (linear) × APOE4 group × Accuracy + Alzheimer's pathology (AT-Term) + Age at baseline + Sex + Vocabulary + Working memory capacity*, random intercept for participant): interaction of recognition-demand level × accuracy (β = -0.25, t(487) = -6.58, $p < 0.001$); three-way interaction of recognition-demand level × APOE4 group × accuracy (β = -0.28, t(486) = -3.70, $p < 0.001$).

Supplementary Table 10. Linear model of effects on VLMT recognition in older adults

	VLMT recognition accuracy							
Predictors	*Estimates*	*std. Error*	*std. Beta*	*standardized std. Error*	*CI*	*standardized CI*	*Statistic*	*p*
(Intercept)	0.96	0.17	0.26	0.21	0.62 – 1.31	-0.15 – 0.68	5.60	**<0.001**
APOE4 Group [Carrier]	-0.03	0.03	-0.37	0.34	-0.10 – 0.03	-1.06 – 0.33	-1.06	0.294
Alzheimer's pathology	-0.00	0.01	-0.03	0.15	-0.03 – 0.03	-0.34 – 0.28	-0.20	0.842
Age at baseline	-0.02	0.04	-0.08	0.14	-0.11 – 0.06	-0.36 – 0.20	-0.57	0.572

Sex [Male]	-0.03	0.03	-0.37	0.28	-0.09 – 0.02	-0.93 – 0.19	-1.34	0.186
Vocab score	-0.00	0.00	-0.01	0.15	-0.00 – 0.00	-0.31 – 0.30	-0.03	0.973
Working memory score	0.00	0.01	0.03	0.15	-0.02 – 0.03	-0.28 – 0.33	0.17	0.862
Observations	57							
R^2 / R^2 adjusted	0.083 / -0.029							

Model: *Verbal Learning and Memory Test (VLMT) recognition accuracy ~ APOE4 group + Alzheimer's pathology (AT-Term) + Age at baseline + Sex + Vocabulary + Working memory capacity*. N = 57. Estimates, standardized betas, 95% confidence intervals, and test statistics are shown for each predictor.

Supplementary Table 11. Linear model of effects on RCFT recognition in older adults

	**RCFT recognition accuracy**							
Predictors	*Estimates*	*std. Error*	*std. Beta*	*standardized std. Error*	*CI*	*standardized CI*	*Statistic*	*p*
(Intercept)	1.00	0.11	-0.08	0.19	0.78 – 1.22	-0.47 – 0.30	9.20	**<0.001**
APOE4 Group [Carrier]	0.01	0.02	0.18	0.32	-0.03 – 0.05	-0.46 – 0.83	0.57	0.572
Alzheimer's pathology	-0.03	0.01	-0.47	0.14	-0.05 – -0.01	-0.76 – -0.18	-3.30	**0.002**
Age at baseline	0.02	0.03	0.11	0.13	-0.03 – 0.08	-0.15 – 0.37	0.87	0.389
Sex [Male]	0.00	0.02	0.08	0.26	-0.03 – 0.04	-0.44 – 0.60	0.30	0.764
Vocab score	-0.00	0.00	-0.22	0.14	-0.00 – 0.00	-0.51 – 0.07	-1.55	0.128
Working memory score	0.01	0.01	0.10	0.14	-0.01 – 0.02	-0.18 – 0.39	0.74	0.462
Observations	57							
R^2 / R^2 adjusted	0.208 / 0.111							

Model: *Rey Complex Figure Test and Recognition Trial (RCFT) recognition accuracy ~ APOE4 group + Alzheimer's pathology (AT-Term) + Age at baseline + Sex + Vocabulary + Working memory capacity*. N = 57. Estimates, standardized betas, 95% confidence intervals, and test statistics are shown for each predictor.

Supplementary Table 12. Linear model of effects of recall-demand level on recall score in older adults

	Recall score							
Predictors	*Estimates*	*std. Error*	*std. Beta*	*standardized std. Error*	*CI*	*standardized CI*	*Statistic*	*p*
(Intercept)	0.04	0.03	0.29	0.15	-0.02 – 0.09	-0.02 – 0.59	1.33	0.190
Recall-demand level	-0.01	0.00	-0.32	0.04	-0.01 – -0.00	-0.41 – -0.23	-7.11	**<0.001**
APOE4 Group [Carrier]	0.01	0.00	0.25	0.25	-0.00 – 0.02	-0.25 – 0.75	1.02	0.314
Alzheimer's pathology	-0.00	0.00	-0.15	0.11	-0.01 – 0.00	-0.37 – 0.08	-1.31	0.197
Age at baseline	-0.01	0.01	-0.16	0.10	-0.02 – 0.00	-0.36 – 0.04	-1.57	0.123
Sex [Male]	-0.01	0.00	-0.75	0.20	-0.02 – -0.01	-1.15 – -0.34	-3.70	**<0.001**
Vocab score	0.00	0.00	0.00	0.11	-0.00 – 0.00	-0.22 – 0.23	0.03	0.976
Working memory score	0.00	0.00	0.24	0.11	0.00 – 0.01	0.02 – 0.46	2.19	**0.033**
Recall-demand level × *APOE4* Group [Carrier]	0.00	0.00	0.00	0.09	-0.00 – 0.00	-0.18 – 0.18	0.01	0.992
**Random Effects**								
σ^2	0.00							
$\tau_{00\ participant}$	0.00							
ICC	0.74							
$N_{participant}$	56							
Observations	110							
Marginal R^2 / Conditional R^2	0.378 / 0.838							

Model: *Recall score ~ Recall-demand level (linear) × APOE4 group + Alzheimer's pathology (AT-Term) + Age at baseline + Sex + Vocabulary + Working memory capacity*. N participant = 56. Estimates, standardized betas, 95% confidence intervals, and test statistics are shown for each predictor. One *APOE4* carrier was excluded as an outlier with values > 2 standard deviations from the mean (gist recall score > 0.083 and detail recall score > 0.054).

Supplementary Table 13. Linear model of effects of *APOE4* group on recall error score in older adults

	Recall error score (log transformed)											
Predictors	*Estimates*	*std. Error*	*std. Beta*	*exp Beta*	*% change*	*standardized std. Error*	*CI*	*standardized CI*	*Statistic*	*std. Statistic*	*p*	*std. p*
(Intercept)	-0.52	1.45	0.54	0.596	-40.40	0.09	-3.43 – 2.39	0.37 – 0.71	-0.36	6.30	0.722	**<0.001**
APOE4 Group [Carrier]	-0.08	0.27	-0.06	0.925	-7.55	0.15	-0.62 – 0.46	-0.36 – 0.24	-0.29	-0.39	**0.772**	**0.699**
Alzheimer's pathology	-0.03	0.12	-0.02	0.967	-3.33	0.07	-0.27 – 0.20	-0.16 – 0.11	-0.29	-0.38	0.770	0.706
Age at baseline	0.47	0.37	0.08	1.60	60.30	0.06	-0.26 – 1.21	-0.04 – 0.20	1.29	1.26	0.203	0.213
Sex [Male]	0.12	0.22	0.09	1.13	12.50	0.12	-0.32 – 0.55	-0.15 – 0.33	0.54	0.77	**0.588**	**0.447**
Vocab score	-0.00	0.01	-0.02	0.998	-0.24	0.12	-0.32 – 0.55	-0.15 – 0.33	0.54	0.77	**0.588**	**0.447**
Working memory score	-0.31	0.12	-0.17	0.736	-26.40	0.06	-0.03 – 0.02	-0.15 – 0.11	-0.19	-0.31	**0.849**	**0.754**
Observations	54											
R^2 / R^2 adjusted	0.199 / 0.095											

Model: *Recall error rate ~ APOE4 group + Alzheimer's pathology (AT-Term) + Age at baseline + Sex + Vocabulary + Working memory capacity*. N = 54. Estimates, standardized betas, 95% confidence intervals, and test statistics are shown for each predictor. Regression coefficients (β) are additionally back-transformed via exponentiation (exp(β)) with corresponding percent (%) change, for interpretation on the original scale. Three older individuals (one *APOE4* carrier, two *APOE4* non-carriers) were excluded from this analysis, as there were methodological problems with their recall error scoring.

References (Supplementary Material)

Bird, C. M., Keidel, J. L., Ing, L. P., Horner, A. J., & Burgess, N. (2015). Consolidation of Complex Events via Reinstatement in Posterior Cingulate Cortex. *Journal of Neuroscience*, *35*(43), 14426–14434.

Burke, A., Heuer, F., & Reisberg, D. (1992). Remembering emotional events. *Memory & Cognition*, *20*(3). https://doi.org/10.3758/bf03199665

Farrer, L. A., Cupples, L. A., Haines, J. L., Hyman, B., Kukull, W. A., Mayeux, R., Myers, R. H., Pericak-Vance, M. A., Risch, N., & van Duijn, C. M. (1997). Effects of Age, Sex, and Ethnicity on the Association Between Apolipoprotein E Genotype and Alzheimer Disease: A Meta-analysis. *JAMA*, *278*(16), 1349–1356. https://doi.org/10.1001/jama.1997.03550160069041

Wessel, I., Zandstra, A. R. E., Hengeveld, H. M. E., & Moulds, M. L. (2015). Collaborative recall of details of an emotional film. *Memory (Hove, England)*, *23*(3), 437–444.
